# Widespread Presence of Human *BOULE* Homologs among Animals and Conservation of Their Ancient Reproductive Function

**DOI:** 10.1371/journal.pgen.1001022

**Published:** 2010-07-15

**Authors:** Chirag Shah, Michael J. W. VanGompel, Villian Naeem, Yanmei Chen, Terrance Lee, Nicholas Angeloni, Yin Wang, Eugene Yujun Xu

**Affiliations:** Division of Reproductive Biology Research, Department of Obstetrics and Gynecology, and Center for Genetic Medicine, Northwestern University Feinberg School of Medicine, Chicago, Illinois, United States of America; University of Washington, United States of America

## Abstract

Sex-specific traits that lead to the production of dimorphic gametes, sperm in males and eggs in females, are fundamental for sexual reproduction and accordingly widespread among animals. Yet the sex-biased genes that underlie these sex-specific traits are under strong selective pressure, and as a result of adaptive evolution they often become divergent. Indeed out of hundreds of male or female fertility genes identified in diverse organisms, only a very small number of them are implicated specifically in reproduction in more than one lineage. Few genes have exhibited a sex-biased, reproductive-specific requirement beyond a given phylum, raising the question of whether any sex-specific gametogenesis factors could be conserved and whether gametogenesis might have evolved multiple times. Here we describe a metazoan origin of a conserved human reproductive protein, BOULE, and its prevalence from primitive basal metazoans to chordates. We found that *BOULE* homologs are present in the genomes of representative species of each of the major lineages of metazoans and exhibit reproductive-specific expression in all species examined, with a preponderance of male-biased expression. Examination of *Boule* evolution within insect and mammalian lineages revealed little evidence for accelerated evolution, unlike most reproductive genes. Instead, purifying selection was the major force behind *Boule* evolution. Furthermore, loss of function of mammalian *Boule* resulted in male-specific infertility and a global arrest of sperm development remarkably similar to the phenotype in an insect *boule* mutation. This work demonstrates the conservation of a reproductive protein throughout eumetazoa, its predominant testis-biased expression in diverse bilaterian species, and conservation of a male gametogenic requirement in mice. This shows an ancient gametogenesis requirement for *Boule* among Bilateria and supports a model of a common origin of spermatogenesis.

## Author Summary

While sexual reproduction is widespread among animals, it remains enigmatic to what extent sexual reproduction is conserved and when sex-specific gametogenesis (spermatogenesis and oogenesis) originated in animals. Here we demonstrate the presence of the reproductive-specific protein Boule throughout bilaterally-symmetric animals (Bilateria) and the conservation of its male reproductive function in mice. Examination of *Boule* evolution in insect and mammalian lineages, representing the Protostome and Deuterostome clades of bilateral animals, failed to detect any evidence for accelerated evolution. Instead, purifying selection is the major force behind *Boule* evolution. Further investigation of *Boule* homologs among Deuterostome species revealed reproduction-specific expression, with a strong prevalence of testis-biased expression. We further determined the function of a deuterostomian *Boule* homolog by inactivating *Boule* in mice (a representative mammal, a class of Deuterostomes). Like its counterpart in *Drosophila* (a representative of the opposing Protostome clade), mouse *Boule* is also required only for male reproduction. Loss of mouse *Boule* prevents sperm production, resulting in a global arrest of spermatogenesis in remarkable similarity to that of *Drosophila boule* mutants. Our findings are consistent with a common origin for male gametogenesis among metazoans and reveal the high conservation of a reproduction-specific protein among bilaterian animals.

## Introduction

Evolution of sexual reproduction, consisting of the origin and maintenance of sex, has been a central focus of evolutionary biology since the time of Darwin. The origin of sex has generally been simplified to the question of the origin of meiosis, which is known to have a single origin among all eukaryotes [1], [2]. However, sexual reproduction in higher eukaryotes is more complex than meiosis alone, and has evolved independently in plants and animals. The fundamental component of animal sexual reproduction is gametogenesis, the differentiation of sexually dimorphic male sperm and female eggs. Unlike meiosis, which is required in both males and females, most other components of gametogenesis are sex-specific or sex-biased, such as sperm tail formation. These traits are subject to strong selective pressures from natural selection, sexual selection, and/or sexual antagonism [3], [4]. Because of these selective forces, sex-biased reproductive-specific traits are known to diverge rapidly. Such patterns of rapid divergence are not only prevalent among morphological traits like male genitalia, but also extend to the molecular level, including DNA sequences, the expression profiles of sex-biased reproductive genes and regulatory pathways underlying sex determination. [5]–[17].

What remains unknown is to what extent features of sexual reproduction can be conserved. Most animals produce sex-specific gametes distinct in size, morphology, motility and development. Animal sperm are predominantly small and motile, with compact nuclei and often a beating flagellum, which are produced through a series of male-specific developmental steps called spermatogenesis. Eggs are usually large in size and immotile, and are produced through a distinct developmental process called oogenesis. The evolutionary origin of such dimorphic features of animal sexual reproduction is intriguing yet difficult to address experimentally since they left no trace in the fossil record. However, the identification of conserved male or female-specific gametogenic proteins across large evolutionary distances could uncover molecular traces of any ancient gametogenic machinery, providing evidence for a common origin of sexually dimorphic traits among animals. While reproductive proteins with conserved homologs in different phyla are not uncommon, most of them are involved in general cellular functions, and are hence also required for other non-reproductive processes [18], [19]. Their sequence conservation likely results from their pleiotropic functional constraints (i.e. additional functions in non-reproductive tissues) rather than their reproductive functions. This is consistent with a model in which the pre-existing somatic cell machinery underwent reproductive specialization during the evolution of gametogenesis in multi-cellular ancestors. A few reproductive-specific proteins have more restricted roles in sexual reproduction in distant species. However they are either required in different sexes or different stages of sexual reproduction in distant lineages of metazoans, or functional information is only available for one metazoan lineage [20]–[25]. Furthermore, most of these proteins appear to be associated with common features of germ cells in both sexes, suggesting that the unique functions that differentiate germ cells from somatic cells are likely to be conserved in animals [26]–[29]. Like meiosis, germ cells are common to both sexes and are therefore not subjected to as strong selective pressures as sex-specific or sex-biased processes are.

Besides meiosis and the specification of germ cells, most of the other components of gametogenesis appear to be sex-biased or sex-specific. Despite a large number of sex-specific gametogenesis proteins uncovered in the model organisms *Drosophila*, *C. elegans* and mice, no conserved male- or female-specific gametogenic factors common to all lineages of animals have been clearly demonstrated [30]–[33]. However, the major steps of dimorphic gametogenesis among animals are very similar. For example, the major steps in spermatogenesis consist of the development of germline stem cells, mitotic proliferation of spermatogonial cells, preparation for and entry into meiosis, meiotic divisions, and finally differentiation of haploid spermatids into highly specialized motile sperm. Most, if not all, of the developmental steps and the developmental sequence of those steps are similar among animals from different phyla [32], [34]. These similarities raise the question of whether they evolved independently in different lineages by convergent evolution, or evolved from a single ancestral prototype. While the absence of a universal male- or female-specific reproductive factor is predictable due to the fast divergence of reproductive proteins and hence is compatible with the hypothesis of multiple origins of spermatogenesis and oogenesis, it does not exclude the alternative single-origin hypothesis. It remains possible that spermatogenesis and oogenesis each evolved from a single prototype, followed by the rapid divergence of most components of the reproductive machinery. However, a few core components of the ancient prototype critical for sperm or egg production could remain conserved.

Identification of such conserved core components is the key to distinguishing between these two possibilities. Such ancient core reproductive components should fulfill the following criteria: present in most, if not all, of the major lineages of animals undergoing sexual reproduction; originated around the time when gametogenesis likely evolved; demonstrated conservation at the sequence, expression and functional levels in species from diverse animal phyla. The most stringent criteria for a conserved male or female gametogenesis factor requires a clear demonstration that these conserved components are only required for gametogenesis in one sex among animals, and not for any other processes, thus excluding any possibility that such factors are conserved due to essential functions outside of gametogenesis. A strong candidate for a conserved male gametogenic factor in animals appears to be *BOULE*, the ancestral member of the human *DAZ* gene family. The human *DAZ* gene family consists of a Y-linked *DAZ* gene and the autosomal *DAZ-Like* (*DAZL*) and *BOULE* genes, all of which share a conserved RNA recognition motif (RRM) and a more divergent DAZ repeat consisting of 24 amino acids rich in N, Y, and Q residues [35]. All DAZ family proteins studied so far appear to be restricted to reproduction [35], [36], and the *DAZ* gene is commonly deleted in men with few or no sperm [37], [38]. Although no mutations in *DAZL* or *BOULE* have been shown to be responsible for infertility in men or women, homologs of *DAZL* and *BOULE* are required for fertility in other species, and over-expression of DAZ family proteins promote the differentiation of human embryonic stem cells towards the germ cell lineage [35], [39]–[43]. Furthermore, a human *BOULE* transgene rescued partial testicular defects of fly *boule* mutants, suggesting functional similarity between these two distant homologs [44].

However, the only two metazoan *Boule* homologs whose function has been characterized in depth revealed opposite gametogenic requirements [39], [40]. Loss-of-function phenotypes of *Boule* homologs in *Drosophila* and *Caenorhabditis elegans* reveal their divergent roles in reproduction, with *boule* required for male reproduction in flies and for oogenesis in the worm [39], [40]. The prevalence of *Boule* homologs among other metazoan phyla remains unexplored, raising the possibility that *Boule* may have undergone adaptive evolution like many other reproductive genes and subsequently diverged at the functional level among different metazoan branches. While partial rescue of the fly *boule* mutant defect by a human *BOULE* transgene suggests functional conservation, this may only reflect similar biochemical properties of all *DAZ* family members [45]. Indeed, frog *Dazl* is able to partially rescue the *Drosophila boule* mutant, despite the fact that frog *Dazl* performs a reproductive function distinct from fly *boule* [46], [47]. We sought to gain further insight into the metazoan evolution of *Boule* and to determine if it has a general conserved reproductive function, or also a conserved sex-specific function. We systematically examined the prevalence of *Boule* homologs in major animal phyla and also the molecular evolution of *Boule* in two distant bilaterian classes. To understand the functional evolution of bilaterian *Boule*, we surveyed the expression of *Boule* homologs in representative bilaterian species and determined the functional conservation of deuterostomian *Boule* through expression and genetic analyses of the mouse *Boule* homolog.

## Results

### Prevalence of *Boule* homologs in metazoans

We first asked what other animal lineages might have *Boule* homologs besides insects, mammals, and nematodes. In order to distinguish *Boule* from homologs of other *DAZ* family members as well as other general RNA binding proteins, we established the signature features of *Boule* that would allow us to identify *Boule* homologs in distant lineages with confidence. We separately aligned the protein sequences of known Boule homologs among two distant metazoan groups, mammals and insects, and established a consensus sequence for the RNA recognition motif (RRM) in each group (Figure S1). To determine general features of the Boule RRM we aligned the mammalian and insect consensus sequences to each other and found a 92-amino acid consensus sequence. The most conserved residues were in the two RNP motifs (PNRI(V)FVGG for RNP2 and DRAGV(I)SKGYGFV(I) for RNP1) that are known to be important for RNA binding in RRM proteins [48]. We also established a consensus sequence for the closely related Dazl RRM, and found it to be distinct from the Boule consensus sequence (Figure S2). Dazl homologs contain slightly different consensus sequences for both RNP2 (VFVGGI) and RNP1 (KGYGFVSF), have distinct sequences surrounding the RNPs, and have a conserved deletion of two amino acids (Figure S2) [35]. Interestingly, the mammalian Boule proteins appeared to share higher sequence similarity than insect homologs despite the fact that mammals have an additional Boule-like protein, Dazl, suggesting that the presence of *Dazl* did not relieve the selective pressure on *Boule* in any significant way. Not only is the sequence of the RRM highly conserved, but the proteins are similar in size, usually around 30 kDa, and contain a single RRM domain near the N-terminus [48]. While it is impossible to align all the exon-intron boundaries due to the extensive genomic divergence between distant species, we found that exon-intron structures spanning the region of the highly conserved RRM (exons 2, 3, 4, and 5) are conserved, except that *Drosophila* exons 3 and 4 are fused into a single exon (Figure S1C). Thus, comparison of the mammalian and insect *Boule* genes reveals conservation not only in specific protein sequences, but also in aspects of the genomic structure underlying these sequences.

**Figure 1:**
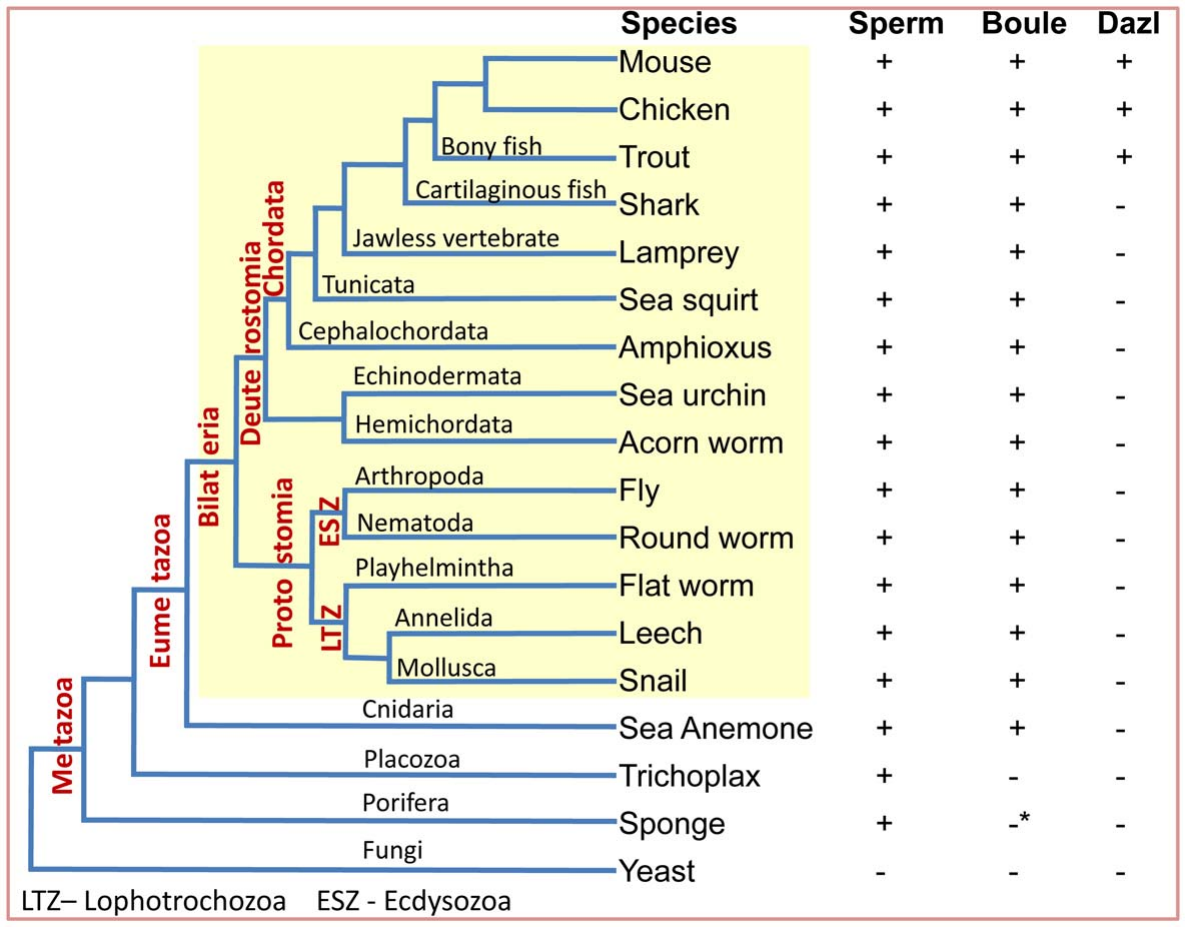
Phylogenetic distribution of motile sperm, *Boule* and *Dazl* homologs among species from major lineages of the animal kingdom and fungi. Sexual reproduction in which male animals produce motile sperm often with flagellum is found in all major phyla of metazoan animals [84]. Representative species of each major metazoan taxon (mostly phylum) were analyzed for the presence or absence of *Boule* and *Dazl* homologs. The common names for those species are listed with the names of the phyla or taxonomical groups they represent printed above their branches. Major superphyla groups and Chordata phylum are labeled vertically near the roots of their phylogenetic branches and bilaterian lineages (Bilateria) are highlighted in yellow. LTZ – Lophotrochozoa, ESZ – Ecdysozoa, Trout—rainbow trout (*Oncorhynchus mykiss*), Shark—elephant shark (*Callorhinchus milii*). All other species names are listed in supplementary information. *The absence of a *Boule* homolog in sponge is tentative since it is based on the draft genome of *Amphimedon queenslandica* (http://www.jgi.doe.gov/sequencing/statusreporter/psr.php?projectid=16318).

**Figure 2:**
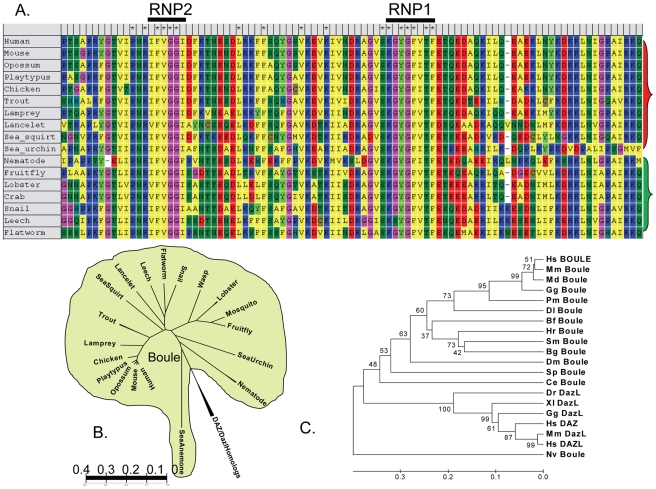
Conservation and prevalence of Boule proteins among animals. (A) Alignment of Boule RRM domains from representative species of both deuterostomes and protostomes reveals conservation of the RNA binding domain, in particular in the regions surrounding RNP2 and RNP1 as well as the C-terminal region. Asterisks (*) mark amino acids conserved in Boule homologs from all species. The red bracket on the right indicates deuterostomian species and the green bracket protostomian species. Colors for individual amino acids are assigned based on similar properties of residues (Table S5). (B) Phylogenetic treatment of homologs from major metazoan branches of human *DAZ* gene family members (*BOULE*, *DAZL* and *DAZ*) using conserved RRM domains. Consistent with being the most ancient member of the *DAZ* family, the *Boule* clade is much more widespread and divergent, including members of the major phyla of protostomes and deuterostomes, while all *DAZ/DAZL* homologs are clustered together in one branch. The *Dazl* homolog clad and *DAZ* clad are much smaller and are restricted to vertebrates or primates, respectively. (C) Rooted tree showing the evolutionary distance among different homologs of the *DAZ* family. The numbers indicate bootstrap values. The evolutionary tree is drawn to scale, with branch lengths in units representing the number of amino acids substituted per site. Hs-*Homo sapiens*, Mm-*Mus musculus*, Md-*Monodelphis domestica*, Gg-*Gallus gallus*, Pm-*Petromyzon marinus*, Dl-*Dicentrarchus labrax*, Bf-*Branchiostoma floridae*, Hr-*Helobdella robusta*, Sm-*Schistosoma mansoni*, Bg-*Biomphalaria glabrata*, Dm-*D. melanogaster*, Sp-*Strongylocentrotus purpuratus*, Ce-*C. elegans*, Dr-*Danio rerio*, Xl-*Xenopus laevis*, Nv-*Nematostella vectensis*.

Since sex-biased genes often undergo lineage-specific loss during evolution [13], we assessed the prevalence of *Boule* homologs in each branch of metazoan evolution (Figure 1). Starting with the Boule RRM consensus sequence, we used Tblastn to search the genomes of species from major phyla representing the two clades of Bilaterians, deuterostomes and protostomes, for *Boule* homologs. Among deuterostomes, Boule homologs were identified in at least one species of every phylum (Figure 1): in Chordata (human, *Homo sapiens*; mouse, *Mus musculus*; chicken, *Gallus gallus*; rainbow trout, *Oncorhynchus mykiss*; elephant shark, *Callorhinchus milii*; lamprey, *Petromyzon marinus*;), Tunicata (sea squirt, *Ciona intestinalis*), Cephalochordata (lancelet or amphioxus, *Branchiostoma floridae*), Echinodermata (sea urchin, *Strongylocentrotus purpuratus*), and Hemichordata (Acorn worm, *Saccoglossus kowalevskii*). Boule homologs were present in many protostomian species of the Ecdysozoa and Lophotrochozoa superphyla (Figure 1, ESZ and LTZ, Figure 2A). Boule was found in fruit flies (*D. melanogaster*), mosquitoes (*Anopheles gambiae*), lobster (*Homarus americanus*), green shore crab (*Carcinus Maenas*), wasp (*Nasonia vitripennis*) and nematodes (*C. elegans*), representing the Arthropoda and Nematoda phyla (Figure 1, ESZ), and also in each phylum of the Lophotrochozoans such as Platyhelmintha (flatworm, *Schistosoma japonicum*), Annelida (leech, *Helobdella robusta*), and Mollusca (snail, *Biomphalaria glabrata*) (Figure 1, LTZ). Therefore, homologs of a known gametogenic protein—Boule—are present throughout both deuterostomes and protostomes.

### Origin of *Boule* in early animal evolution

Next, we asked when *Boule* arose during evolution by determining whether *Boule* homologs are present in basal, non-bilaterian metazoans or beyond the animal kingdom in plants or fungi.

**Figure 3:**
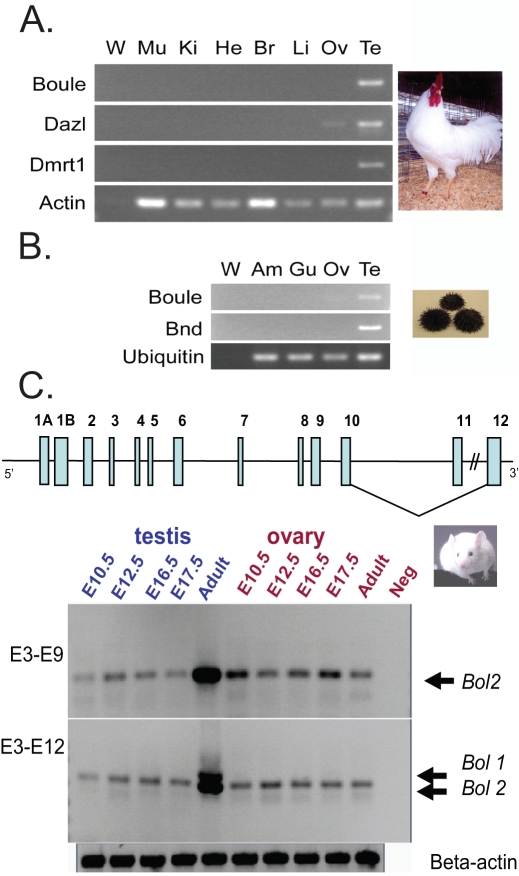
Conservation of reproduction-specific and testis-enriched expression of three deuterostome *Boule* homologs. (A) RT-PCR survey using chicken *Boule* and *Dazl* specific primers on different tissues from adult rooster and hen (*Gallus gallus*). *Dmrt1* (*Doublesex and mab-3 related transcription factor 1*) is a testis-specific gene and actin was used as a loading control. (B) RT-PCR survey using sea urchin-specific primers on different tissues from purple sea urchin (*Strongylocentrotus purpuratus*). *Bnd* (*Bindin*) is a testis-specific gene and ubiquitin was used as a loading control. W, water; Mu, muscle; Ki, kidney; He, heart; Br, brain; Li, liver; Ov, ovary; Te, testis; Am, Ampullae; Gu, guts. (C) Mouse *Boule* consists of 13 exons that encode multiple isoforms resulting from 5′ and 3′ alternative splicing. Two predominant mouse *Boule* isoforms – *Bol1* and *Bol2* – are male-biased and are restricted to or enriched in adult testes. *Bol1* contains all 12 exons whereas *Bol2* contains all exons except exon 11. RT-PCR analysis of RNA from mouse embryonic and adult gonads using primers corresponding to either exons 3 and 9 (upper panel, 501-bp band) or exons 3 and 12 (lower panel, 847-bp and 712-bp bands) reveals that full-length *Bol1* is only expressed in postnatal testes, while *Bol2* lacking exon 11 is also detectable at low levels throughout gonadal development in addition to its high adult testis expression. A similar pattern was confirmed using primers for exons 1 and exon 12.

Based on the consensus *Boule* features, we determined that *Boule* homologs are absent in fungi and plants, suggesting that *Boule* is restricted to the animal lineage (Figure S3A, Figure 1). We then explored the genomes of basal metazoan animal species and found that there is no *Boule* homolog in the most primitive animal, *Trichoplax* (Figure S3B, Figure 1). However, we identified a *Boule* homolog in the sea anemone, a species from the primitive Cnidaria phylum. Comparison of the consensus *Boule* sequence against the sea anemone genome (*Nematostella vectensis*) reveals two proteins with high similarity [49]. Surprisingly, the RRMs of both proteins contain characteristics of the Boule consensus sequence, while one of the sea anemone proteins has identical signature RNP1 and RNP2 motifs (PNRIFVGG and GVSKGYGSVT) to those of the Boule consensus domain (Figure 3C). Furthermore, unlike the fused exons 3 and 4 in the *Drosophila boule* genomic structure, the sea anemone *boule* gene has separate exons 3 and 4 as in humans, suggesting that the ancestral *Boule* gene contained separate exons 2, 3, 4 and 5 that encoded the RRM. This gene (XM_001637198) is predicted to encode a protein around 22 kDa, close to the typical size of Boule proteins. Hence, a *Boule* homolog is present in the sea anemone, a representative of Cnidaria (Figure S3C, Figure 1). A second sea anemone protein also has some similarity to the characteristic Boule RNP1 and RNP2, but there are multiple differences in critical positions and a greater divergence from the Boule consensus sequence (Figure S3C). Furthermore, the gene itself does not possess two conserved exon/intron junctions in the second half of the RRM domain that are present in all other species examined, including *Drosophila*. Therefore, the second protein is likely a more divergent duplicate of the ancient *Boule* gene, specific to the Cnidarian lineage.

The sea anemone is one of the most primitive metazoan species that undergoes sexual reproduction. It has separate sexes, inducible spawning and external fertilization [49]. Our finding places the origin of the *Boule* gene prior to the divergence of Bilateria from Cnidaria, but likely after *Trichoplax* branched from the common ancestor of eumetazoans, making *Boule* one of the few ancient animal gametogenic proteins known so far. Further analysis of *Boule* homologs in other basal metazoan lineages could better pinpoint the origin of metazoan *Boule*.

*Dazl* arose through a duplication of *Boule*, likely after protostomian and deuterostomian splitting, but the exact point of *Dazl* origin within deuterostome evolution has not been defined [35]. Homologs of *Dazl* have been identified in mammals, birds, reptiles, amphibians and fish [35], [50], [51], but whether *Dazl* is present in other non-vertebrate deuterostomes is unknown. Using the Dazl RRM domain, we searched for *Dazl* homologs in the genomes of acorn worm from Hemichordata (*Saccoglossus kowalevskii*), sea urchin from Echinodermata (*Strongylocentrotus purpuratus*), lancelet from Cephalochordata (*Branchiostoma floridae*), and sea squirt from Tunicata (*Ciona intestinalis*). We could not detect any canonical *Dazl* homologs (Figure 1). The highest BLAST hit from those genomes were *Boule* homologs, suggesting that *Dazl* is not present in either non-chordate deuterostomes or primitive chordates, and is likely restricted to the vertebrate lineage. To further determine the origin of *Dazl* in vertebrate evolution, we searched the genomes of the jawless fish, lamprey (*Petromyzon marinus*) and could not identify a *Dazl* homolog (http://genome.wustl.edu/genomes/view/petromyzon_marinus/) (Figure 1). Given that *Dazl* is present in bony fish such as zebrafish and medaka [52], [53], we then asked if *Dazl* is present in the cartilaginous fish, phylogenetically the oldest group of living jawed vertebrates. We searched the genome of the elephant shark (*Callorhinchus milii*) and found no evidence of a *Dazl* homolog, though a shark *Boule* homolog is present [54]. This analysis suggests that *Dazl* originated around the time of vertebrate radiation, likely in the ancestral lineage of bony fish (Figure 1).

To further determine the evolutionary relationship of metazoan *Boule* homologs, we performed phylogenetic analysis of *Boule* homologs from the major animal branches, together with homologs of the other members of the *DAZ* family, *Dazl* and *DAZ* (Figure 2B and 2C). *Boule* clearly represents the most ancient and widespread clade among the *DAZ* family members, present from sea anemone to human, whereas all *Dazl* and *DAZ* homologs can be clustered together in one branch. This is consistent with the distinct reproductive functions of *DAZ* and *Dazl* homologs, and the late arrival of *Dazl* in vertebrate evolution and *DAZ* in primate evolution (Figure 2B and 2C) [35], [38], [41], [47], [55]. Conservation of *Boule* homologs in major lineages of animals and their evolutionary relationship throughout animal evolution suggests that *Boule* is a fundamental component of eumetazoan reproductive machinery essential for the survival of most animal species.

### Purifying selection, not positive selection, is predominant in the molecular evolution of *Boule*

The ancient origin and widespread presence of such a reproductive gene is in stark contrast with the pervasive rapid evolution usually associated with reproductive genes, especially male reproductive genes [5], [12]. The presence of *Boule* in various animals provided the rare opportunity to examine how selective forces shaped the molecular evolution of a reproduction-specific gene in distant lineages. We therefore examined *Boule* homologs that recently diverged from each other for any signs of adaptive evolution. We analyzed two separate groups of homologs to determine if *Boule* is under different selective pressure when *Dazl* homologs are present. We compared the entire *boule* coding sequences among seven *Drosophila* species (*D. melanogaster*, *D. sechellia*, *D. yakuba*, *D. virilis*, *D. erecta*, *D. willistoni* and *D. ananassae*) as well as eight representative mammalian species [56].

To determine if positive selection has played a role in *Boule* evolution, we compared the ratio of the rate of nucleotide changes that result in a non-synonymous amino acid substitution (Ka) to the rate of nucleotide changes that cause a synonymous amino acid substitution (Ks). Positive selection is a process that favors the retention of mutations that are beneficial to the reproductive success of an individual. Neutral theory predicts that the rate of non-synonymous substitutions (that by definition affect protein sequence) is equal to the rate of synonymous substitutions. If a protein has evolved under positive selection, there are more non-synonymous substitutions (Ka) than synonymous substitutions (Ks), and an accordingly high Ka/Ks ratio. If the protein evolved under purifying selection or negative selection, a process that removes deleterious alleles, there is a decrease or absence of non-synonymous substitutions, and therefore Ka/Ks is much smaller than that expected under neutral theory. A Ka/Ks greater than 1 is a strong indication of positive selection whereas only a Ka/Ks smaller than 0.1 usually suggests a role of purifying selection.

Among all pairwise comparisons among *Drosophila* species, we found that non-synonymous substitutions (Ka) were not in excess of synonymous substitution (Ks). Instead, Ka/Ks ratios for all pairwise comparisons were below 0.1 (Table S1), significantly lower than the ratio reported for rapidly diverging proteins [10], [12]. Similarly, all pairwise comparisons among mammalian species revealed Ka/Ks ratios below 0.1 (Table S2), indicating that the presence of *Dazl* homologs in mammals had little impact on the selective pressure on *Boule* homologs. Furthermore, this suggests that positive selection was not the major force driving the evolution of *Boule* either in *Drosophila* or mammals. Instead, the low Ka/Ks ratio suggests that purifying selection was responsible for the strong functional constraint on the entire protein, making *Boule* an exception to the rapid evolution commonly seen in reproductive genes [5], [9].

### Male-biased gonadal expression among bilaterian *Boule* homologs

The prevalence and strong functional constraint of *Boule* throughout protostomes and deuterostomes suggests that *Boule* is likely a common reproductive factor with a critical function essential for the survival of bilaterian species. However the only *Boule* homologs functionally characterized exhibit divergent roles in reproduction, with *Drosophila boule* necessary for male reproduction and the *C. elegans boule* homolog, *daz-1*, required for egg production [39], [40]. Recently, the *Boule* homolog in the fish *Medaka* was reported to be expressed in both testes and ovaries [53]. Such divergent roles and expression during gametogenesis raised the question of what the ancestral function of *Boule* was, and whether the expression and function of *Boule* homologs might have diverged despite the high conservation of the functional motif. Since *Boule* function has only been examined in protostomes (*C. elegans* and *Drosophila*), we reasoned that by determining *Boule* expression patterns in deuterostomes we could ascertain whether or not the expression or function of *Boule* is conserved among bilaterians.

We chose two deuterostome species (chicken and sea urchin) from separate phyla and asked if *Boule* homologs are preferentially expressed in the testis or ovary. Like *Drosophila* and *C. elegans*, the sea urchin is also an invertebrate and has only the *Boule* gene, whereas chicken is a vertebrate with both *Boule* and *Dazl*. We identified homologs of *Boule* in chicken (*G. gallus*) and purple sea urchin (*S. purpuratus*) (Figure 1, Figure 2A) [51], [57], and found that chicken *Boule* is expressed specifically in the testis, and is not present in ovaries or any other organs we examined (Figure 3A, Figure S4). However, chicken *Dazl* is expressed in both testes and ovaries, similar to mammalian *Dazl* [41], [58]. The expression of the *Boule* homolog in sea urchin, a primitive deuterostomian species from the Echinodermata phylum, is also testis-biased and not expressed in any non-gonadal tissue (Figure 3B). A transcript that lacks a complete RRM domain was detectable at low levels in ovary (not shown). However, this ovarian transcript may not be functional and is likely the isoform previously reported in sea urchin ovary and eggs by *in situ* hybridization [57]. Together these results show that deuterostome homologs of *Boule* are also reproduction specific, like their protostome counterparts, but with a tendency toward testis-biased expression.

Since the nematode *Boule* homolog is only required for ovarian function but not male gametogenesis, and *Boule* transcripts have been detected in the ovaries as well as in the testes of some other species [53], [57], [59], we wondered if such ovarian expression in sporadic species is a lineage-specific phenomenon or if it is a common feature. Thus, we turned to the laboratory animal model, the mouse, for an in-depth gene expression and functional analysis. Although mammalian *Boule* is highly expressed in the adult mouse testis but not ovary, it is not known if *Boule* is expressed in the ovary during development [35]. In view of the different timing of meiotic initiation in female and male mammals, we determined the developmental expression profile of mouse *Boule* during both male and female embryonic gonadal development (embryonic day 10.5, E12.5, E16.5 and E17.5) in comparison with adult gonads.

We first characterized the entire mouse *Boule* genomic region and identified alternatively spliced isoforms (Text S1 and Figure 3C). Using primers spanning all 12 exons, we found two major *Boule* transcripts, both of which were most highly expressed in the adult testis. The primary *Boule* transcript contained all 12 exons (Bol1) and was expressed only in the adult testis, whereas a second transcript lacking exon 11 (Bol2) was highly expressed in adult testes but also detectable at low levels in early embryonic gonads of both sexes and the adult ovary (Figure 3C). We thus confirmed that the predominant expression of *Boule* during reproductive development is in the adult testis, including a testis-specific isoform, and also identified previously unreported low levels of *Boule* RNA in mouse ovaries. Together with previous findings, a total of seven out of eight bilaterian species examined (human, mouse, cattle, chicken, fish medaka, sea urchin and fruit fly) representing three different phyla express *Boule* in the adult testis [35], [53], [60]. Expression is in the same cell types (spermatocytes and spermatids) in the testes of the human, mouse and fish medaka, suggesting conservation of developmentally-regulated testicular expression of *Boule* in vertebrate animals [35], [53].

The observation that *Boule* homologs show predominantly testis-biased expression in diverse species is consistent with a conserved male gametogenic function in bilateral animals. However, the oogenic requirement seen in *C. elegans* taken together with detectable levels of ovarian expression in several species suggests the possibility that an additional oogenic function is also conserved. Alternative *Boule* transcripts detected in mouse ovaries or embryonic gonads, albeit at much lower levels, could still play an important physiological function and therefore contribute to its sequence conservation. To ascertain if *Boule* is functionally conserved in deuterostomes and if ovarian expression of *Boule* is physiologically significant, it is necessary to examine the physiological function of *Boule* in deuterostomes.

### Generation of a deuterostomian *Boule* mutation

**Figure 4:**
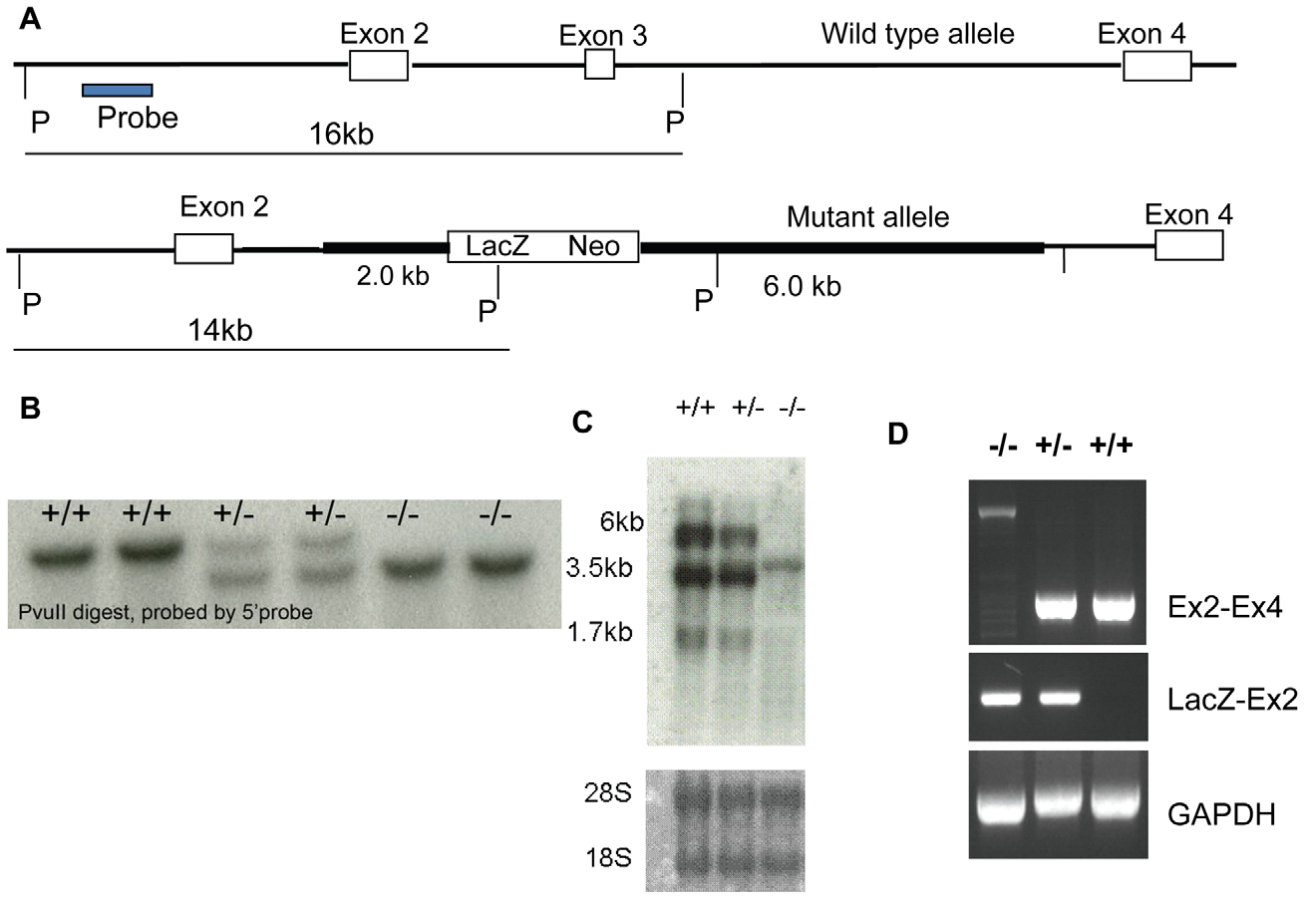
Generation of a loss-of-function *Boule* mutation in mice. (A) Gene targeting strategy with wild type genomic structure from exon 2 to exon 4 (top) and the successfully targeted locus (bottom). Thick bars represent the two genomic regions used for homologous recombination in the gene targeting construct. PvulI (P) restriction sites and the position of the 5′ probe are shown. (B) Southern hybridization with the 5′ probe shows that the wild type contains a 16-kb PvuII fragment, whereas homozygotes contain a 14-kb PvuII fragment and heterozygotes contain both 16-kb and 14-kb fragments, thus confirming homologous recombination. (C) Northern hybridization using *Boule* cDNA shows that all three wild type *Boule* transcripts are absent in the homozygotes; instead a novel transcript corresponding to exon 1, exon 2, and lacZ was detected. (D) RT-PCR using primers spanning exons 2 to 4 further confirmed the complete absence of wild type transcripts. Expected wild type products between exon 2 and 4 were present in heterozygotes and the wild type but absent in homozygotes. The weak amplification of a very large fragment in homozygotes represents the PCR product from the chimerical transcript spanning exon 2, LacZ, and exon 4 of the mutant allele.

To determine if the male-specific requirement of *Drosophila boule* is functionally conserved among Bilateria, we set out to generate a mutation of a deuterostomian *Boule* homolog to investigate its physiological requirement. We used mice as a representative species of deuterostomes, and used gene targeting to delete the RNA binding domain, and thus disrupt the critical function of mouse *Boule* (Figure 4). We replaced exon 3, which encodes a part of the RNA binding domain and is present in all *Boule* isoforms (Figure 3C), with a lacZ-neo vector through homologous recombination in embryonic stem cells. The removal of exon 3 resulted in a deletion of the RNA binding domain and a frame-shift in the remaining transcript. This transcript is expected to produce a truncated BOULE protein missing both its RNA binding domain and the remaining C-terminal portion of the protein. Four chimeric mice were derived and correct homologous recombination as well as germline transmission of the *Boule* mutation was confirmed. Homozygote mice recovered from matings among heterozygotes were identified by genotyping and confirmed by Southern hybridization (Figure 4B).

We next determined if the mouse *Boule* mutation was a complete loss-of-function mutation. We performed Northern blot hybridization on RNA from the testes of wild-type, heterozygote, and homozygote mice with *Boule* cDNA as a probe and found that there are three *Boule* transcripts present in wildtype testes, all of which are absent in homozygous *Boule* mutants. Instead, a single novel transcript corresponding to the size of the predicted chimeric transcript consisting of the truncated *Boule* and beta-geo (a transcript containing exon 1, 2 and lacZ, Figure 4C) is present. We further confirmed the absence of wild type *Boule* transcripts by the more sensitive RT-PCR and did not detect exon 3 in the mutant transcript. Instead we only detected a much larger PCR product spanning exon 2 and 4 in homozygotes (Figure 4D). This large PCR product is absent in wildtype and contains the lacZ gene from the knockout vector. Hence we conclude that *Boule* expression is completely disrupted in the mutant, and we have established a loss-of-function allele in the mouse *Boule* homolog.

### Mouse *Boule* is essential for male fertility but not for viability, growth, or female fertility

**Figure 5:**
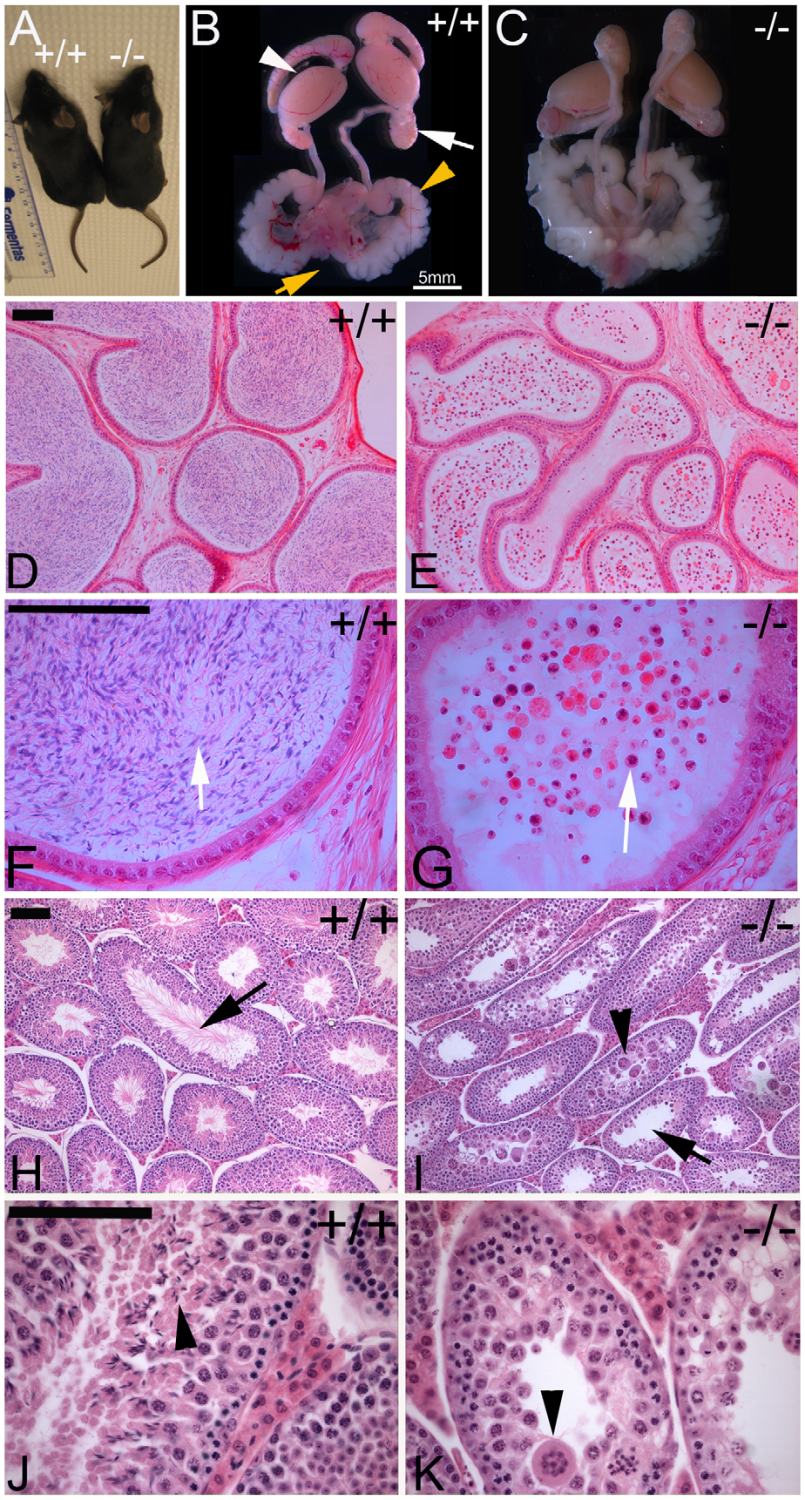
Mouse *Boule* mutant males are infertile, resulting from a global arrest of spermatogenesis. (A) Knockout mice are indistinguishable from those of wild type in morphology and size. (B) The wild type male reproductive tract consists of a pair of oval-shape testes (white arrow head) attached to the epididymis (white arrow), seminal vesicle (yellow arrowhead), and urethra (yellow arrow). (C) The reproductive tract in mouse *Boule* mutants appears normal, similar to that of wild type mice. Wild type epididymis tubules contain lots of motile sperm (D), and condensed sperm nuclei and sperm tails (arrow) are clearly visible (F at higher magnification). In the *Boule* mutant, epididymal tubules lack mature sperm and some are half-empty (E). Instead, small degenerating round cells are found inside the tubules (arrow in G). (H) Testicular sections from wild type mice showing the presence of sperm in the lumen (arrow in H) and elongating spermatids (arrowhead in J). (I, K) Sections from *Boule* mutant testes showing the absence of both sperm from the lumen (arrow in I) and elongating spermatids. Mouse *Boule* mutant testes contain degenerating cysts of germ cells (arrowheads in I and K). The scale bars are equal to 100 µm unless noted.

Homozygote *Boule* mutants exhibited normal viability, growth and mating behavior (Figure 5A). We recovered the expected number of homozygotes from heterozygote matings (wild type∶ heterozygotes∶ homozygotes = 44∶79∶43), indicating that there was no effect on survival. We next tested the fertility of mice homozygous for the *Boule* mutation to determine whether the *Boule* mutation affected male and/or female reproduction. Six homozygote males were each mated with two wild type females and individually produced no pups after four months. In contrast, wild type males sired at least two litters each during that time, suggesting that the homozygous *Boule* males were sterile. Female *Boule* homozygotes showed no obvious defects and were fertile, producing an average of 8.3 pups per litter (8.3±1.4, n = 12) with heterozygote males, similar to wild type or heterozygote females (7.7±2.1; n = 7 for wildtype; 7.6±2.4; n = 18 for heterozygotes). Homozygote females continued to be fertile up to the oldest age tested (12 months). Thus, mutation of mouse *Boule* disrupts male reproduction but does not affect normal development, growth or female fertility, suggesting that mammalian *Boule* is required only for male reproduction, similar to fly *boule* but different from worm *daz-1*.

Similar physiological requirements of *Drosophila* and mouse *Boule* homologs suggest possible conservation of an ancient male gametogenic requirement. However, it is also possible that such similarity is a mere coincidence since out of hundreds of bilaterian species, only homologs from three species are functionally characterized, with one out of the three being functionally divergent. We reasoned that if mouse and *Drosophila Boule* function is conserved, then the specific reproductive defects of the loss-of-function mutations in both species should be more likely to be similar than if they had evolved independently by chance. We therefore determined whether mouse *Boule* and fly *boule* function within similar processes of male reproduction. In both flies and mice, the major reproductive organs are clustered together in the male reproductive tract. The tract consists of a pair of testes for sperm production; a pair of sperm storage/maturation organs (the epididymis in mice and seminal vesicle in flies); accessory glands for providing proteins, other nutrients and seminal fluid that accompany sperm migration and fertilization (prostate, seminal vesicles and coagulating glands in mice and accessory glands in flies); and a sperm transport duct used for sperm transportation and maturation (vas deferens and urethra in mice and ejaculatory duct in flies) (Figure 5B) [30]. While the major components of the male reproductive tract in mammals and insects appear to serve similar reproductive functions, it is not known if any components are evolutionarily related between vertebrates and invertebrates. In mouse *Boule* mutants, all the components of the male reproductive tract are present and intact (compare Figure 5B and 5C), similar to that of the fly *boule* mutant [39]. Compared with wild type mice, the male reproductive tracts of *Boule* homozygous mutant mice were morphologically indistinguishable except for the testes, which are smaller by weight [61]. Hence the sterility defect is the result of a defect in the testis, similar to the sterility defect associated with the *Drosophila boule* mutation [39], [61].

### Mouse *Boule* mutation causes a global arrest of sperm development

Further characterization of the reproductive defects revealed that mouse *Boule* mutant epididymides lacked mature sperm (Figure 5E), and instead contained degenerating cells that were not seen in the wild type (compare Figure 5D and 5F to Figure 5E and 5G). Therefore, the observed male sterility of the *Boule* homozygous mutant mice appears to be due to a complete absence of sperm in the epididymis.

Next, we examined the developmental impact of the mouse *Boule* mutation on sperm production. While the overall testicular structure was normal and all the somatic cell types were present in mouse *Boule* mutants, the effect of mouse *Boule* mutation on sperm development was dramatic, with a complete halt of spermatogenesis inside all seminiferous tubules of the testis. Both mature sperm and developing elongating spermatids were entirely absent from the lumen of individual seminiferous tubules (compare Figure 5H and 5J to Figure 5I and 5K). This indicates that the failure to produce sperm resulted from a major block in sperm production due to a global arrest of spermatogenesis prior to spermatid differentiation, similar to the spermatogenic defect seen in the testes of *boule* mutant flies [39], [61]. Such similar global, spermatogenic-specific impacts of mutations in orthologs in divergent phyla is surprising and unprecedented, given the vastly different organization of testicular structure, type of spermatogenesis and differences in the contribution of hormonal control in mammals and insects.

In *Drosophila*, spermatogenesis is cystic, where a single spermatogonial cell and its clonal descendants are encapsulated in a somatic cyst throughout sperm development. Though mouse spermatogenesis is acystic, we observed prominent ball-like structures containing degenerating cells in the mouse *Boule* mutant, resembling the degenerating cysts seen in the fly *boule* testis (Figure 5I and 5K, arrowheads). Although the cyst structure is not present in mammalian spermatogenesis, descendant cells from a single spermatogonial stem cell remain connected with each other through cytoplasmic bridges during mouse sperm development, similar to that in *Drosophila* spermatogenesis [62]–[65]. This phenomenon could lead to the merging of multiple interconnected arrested spermatogenic cells in *Boule* mutant testes, resulting in such giant “cysts” with multiple nuclei. Despite the distinct modes of spermatogenesis in mice and *Drosophila*, the mouse *Boule* and fly *boule* mutations caused a remarkably similar and specific global arrest of spermatogenesis. Though further characterization of the developmental and cellular defects in mouse *Boule* mutant testes is needed to determine the full extent of similarity in developmental and cellular defects between mouse and *Drosophila Boule* mutants [61], our data demonstrate a key physiological requirement of *Boule* in sperm development and conservation of its male reproduction function between two distant lineages of a protostome (*Drosophila*) and a deuterostome (mouse).

## Discussion

Evolution of reproductive traits and genes is of the utmost interest to our understanding of the central questions in evolutionary biology such as speciation. However, the relatively rapid divergence of sex-biased reproductive genes in comparison with somatic cell proteins or non sex-biased reproductive proteins during evolution has made it difficult to study the evolution of sex-specific reproductive systems across extended evolutionary distances. Even though some reproductive genes are conserved beyond a given phyla, they are often also involved in other developmental processes. Such broad functionality compounds studies of their reproductive evolution because the selective pressures driving their evolution may be due to critical somatic functions, and not a reproduction-related function. The human *DAZ* family of reproductive genes, with homologs in diverse species, many of which are specifically expressed in reproductive tissues, are ideal candidates for the study of reproduction-specific gene evolution [35], [38], [40], [41], [46], [53], [60]. In particular, *Boule*, the ancestral gene member, is reproductive specific in flies and worms.

**Figure 6:**
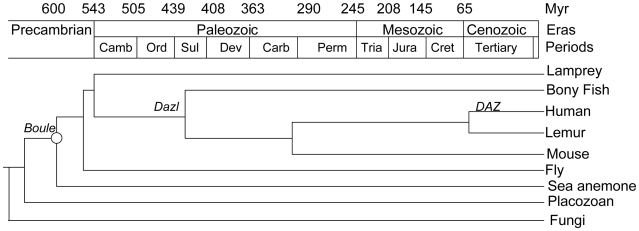
Schematic representation of evolution history of the three *DAZ* family members—*Boule*, *Dazl*, and *DAZ*—during metazoan evolution. The birth of *Boule* is estimated to be after the divergence of placozoan from the common ancestor (open circle) of Cnidaria (such as sea anemone) and Bilateria. *Dazl* arose through duplication from ancestral *Boule* during the evolution of bony fish, possibly after its split from lamprey and cartilaginous fish but prior to the divergence of ray-finned fish and lobe-finned fish (i.e. tetrapod animal lineage). After their divergence from New World monkeys such as lemur, Old World monkey *Dazl* duplicated and gave rise to *DAZ* [55]. Myr –millions of years. The geological scale is based on [67], [85].

**Figure 7:**
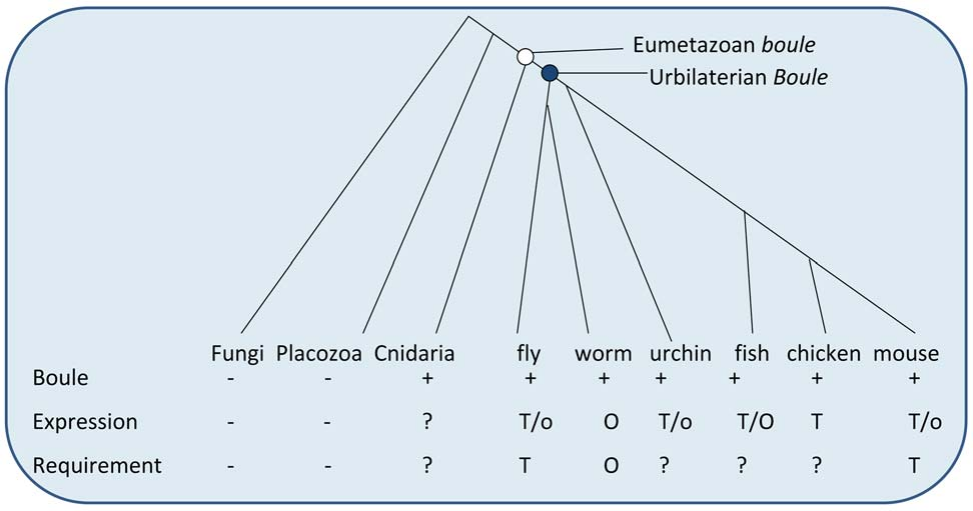
Expression and functional requirement of *Boule* homologs in metazoan species studied. The presence of a *Boule* homolog (+), testis expression or functional requirement (T) or ovary expression or functional requirement (O) is marked. Low level expression in the ovary is marked as a lower-case “o” to distinguish it from abundant ovarian expression, marked as a capital “O”. The information on *Boule* homologs are based on this work and previous reports [35], [39], [40], [53]. The ancient *Boule* gene in the common ancestor of Bilateria (the Urbilaterian) is indicated as a filled circle, and ancestral *Boule* likely originated during the evolution of eumetazoans, shown as an open circle. The ancient bilaterian *Boule* was reproduction-specific, and we hypothesize that it functioned in spermatogenesis, based on a predominance of testis-biased expression in diverse lineages of bilaterian animals and the conservation of a male reproductive function in *Drosophila* and mouse.

We identified homologs of *Boule* in the major phyla of metazoans, reconstructed the evolutionary history of *Boule*, and began to determine its functional divergence. We found that Boule, unlike other reproductive proteins, has been maintained in all major phyla of bilaterian animals as well as in Cnidarians, but are absent in the most primitive animals (the placozoan *Trichoplax*), fungi and plants (Figure 1). We found that *Dazl* homologs are only present in vertebrates, supporting the hypothesis that *Boule* is the ancestral member of the *DAZ* family [35]. *Dazl* homologs were absent in representative species of non-vertebrate deuterostomes and cartilaginous fish (elephant shark), but were present in bony fish and tetrapod animals (Figure 1). This places the origin of *Dazl* after the divergence of bony fish from cartilaginous fish but before the arrival of tetrapod animals (Figure 6). On the other hand, the widespread presence of *Boule* in eumetazoan animals indicates that the ancient *Boule* gene was present as early as 600 million years ago in the Precambrian era, in the common ancestors of Bilaterians (often called Urbilateria) as well as eumetazoans (Figure 6 and Figure 7) [66], [67].

Interestingly, human *BOULE* has previously been shown to be able to function in *Drosophila* testes, and can even rescue meiotic defects of *boule* mutant flies, suggesting a conservation of a spermatogenesis-specific function [35], [44]. However, the *C. elegans boule* homolog *daz-1* is required only in oogenesis [40], making it unclear whether such a transgenic replacement in the fly actually represents a legitimate functional conservation. Furthermore, both *C. elegans* and *Drosophila* are protostomes, so whether *Boule* is even required for reproduction, let alone restricted to spermatogenesis, in any deuterostome species was not known. Using mice as a representative deuterostome, we generated a *Boule* null allele to address this question (Figure 4). *Boule* is required only for male reproduction in mice (Figure 5), similar to insect *boule*, revealing not only a conserved function, but suggesting an ancient requirement of *Boule* in gametogenesis. Furthermore, the requirement of mouse *Boule* for male reproduction and its dispensability for female fertility suggests that low level expression of *Boule* in embryonic germ cells and adult ovaries is not essential for either the development of germ cells or the production of female gametes. Similarly, *Drosophila boule*, initially thought to be testis-specific, has also been found to be alternatively spliced and expressed in the ovary and even some somatic tissues at a low level, though loss-of-function similarly only causes male sterility [59], [68]. Interestingly, this result shows that *Boule* has a spermatogenesis-specific requirement conserved in at least two distant lineages of bilateral animals, making it a strong candidate for a conserved male gametogenesis factor between *Drosophila* and mammals.

Given that mouse *Boule* is required for sperm production like fly *boule* but different from *C. elegans daz-1*, we propose that Urbilaterian *Boule* had an ancestral function in male gametogenesis which was lost during the evolution of the nematode lineage (Figure 7). This is consistent with the higher sequence divergence of the *C. elegans* daz-1 RNA binding motif than most other bilaterian Boule homologs (Figure 2). While we can not rule out the possibility that the similar male gametogenic requirement in mice and *Drosophila* is a coincidence and both evolved independently, the striking similarity in the reproductive defects of loss-of-function mutants of *Drosophila* and mouse *Boule* homologs (male specific infertility, global arrest of spermatogenesis, absence of elongating spermatids and mature sperm) argue against such a possibility. Furthermore, the predominance of testis-biased expression of *Boule* homologs among distinct bilaterian species (Figure 3), supports a model of an ancient male gametogenic function (Figure 7).

It is important to note, however, that this model does not exclude the possibility of an additional ancestral ovarian function of Urbilaterian *Boule*. Since ovary expression of *Boule* is also prevalent among diverse animals, and *C. elegans daz-1* is required in females, the ancestral *Boule* gene may have also played a role in oogenesis, which may have been subsequently lost in specific lineages. Our data does not rule out this possibility, and such an ancestral oogenesis function of *Boule* could be in addition to our proposed ancient spermatogenesis function. Further functional analysis in other lineages, including medaka where strong ovarian *Boule* expression has been observed, could help determine the more likely scenario [53]. Additionally, characterization of *Boule* homolog(s) in the sea anemone, an outgroup to the bilaterian lineage, could provide further insights into the ancestral roles of *Boule*.

Whether or not an ovarian function of *Boule* is also conserved, our discovery that mammalian *Boule* is required only for sperm development like its fly counterpart is the first such demonstration of a conserved spermatogenesis-specific function in both lineages. While spermatogenesis occurs in the testes of different animal lineages, it is not known if either spermatogenesis or the testis itself is evolutionarily related between vertebrates and invertebrates. The fly testis, which is a single tube with a linear progression of spermatogenesis, appears different from the mouse testis, which is composed of many seminiferous tubules with a concentric progression of spermatogenesis from the periphery of the seminiferous tubules towards the lumen. However, if we focus on a single cycle of spermatogenesis within a segment of a mouse seminiferous tubule and compare it with a fly testis tubule, we see similar spermatogenic cell types present inside the fly testis tubule and the mouse seminiferous tubule segment [69]. Spermatogenesis in both species starts with spermatogonial stem cells located in a specific position of the tubule, attached to the apical end in fly and to basement membrane in mice, which move and progress into later stages of cell types in one direction, towards the basal end of the testis tubule in fly and towards the lumen in mice. All the major stages of sperm development appear to be present in both species and arranged in a similar spatial and temporal pattern. If both developmental processes evolved from an ancient primitive spermatogenesis prototype, one would predict the presence of at least some common male gametogenesis-specific regulators in both lineages. Yet no such common male gametogenesis factor has been demonstrated to be required exclusively for sperm production in both lineages. The lack of a universal male reproductive factor among all animal lineages, while consistent with rapid evolution of male reproductive genes, is in contrast to the prevalence of sexual reproduction and in particular to the similarity in male gametogenesis among metazoan animals [34], [69]. This paradox led to the question of whether such similarity in the reproductive traits arose from convergent evolution or from conservation of an ancient prototype in the common ancestor.

Furthermore, male reproductive traits and genes undergo rapid adaptive evolution in diverse lineages such as *Drosophila*, fish, rodents and primates [5], [9]–[12], [16], [17]. Male-biased genes exhibit a higher divergence of expression among closely related species than female-biased genes or genes expressed in both sexes [7], [13]. Additionally, testis-biased genes have the highest rate of extinction and species-specific de novo gene formation during evolution [13], [70], [71]. For example, the most widespread testis-specific proteins among both vertebrates and invertebrates appear to be sperm nuclear basic proteins (SNBP). Many organisms replace histones with a set of small basic structural proteins (SNBP) or protamines to establish a highly compact sperm chromatin structure [72], [73]. Although all metazoan SNBP homologs share their common ancestry with somatic histone H1 protein, the testis-specific SNBPs in different lineages have undergone extensive lineage-specific loss and dynamic evolution, including adaptive evolution [5], [73]. Furthermore it remains unclear if vertebrate and invertebrate protamine homologs are functionally conserved. Loss of one copy of either mouse *Protamine-1* or *Protamine-2* leads to male sterility, but in contrast, fly sperm carrying a deletion of both protamine-like homologs appears to be functional [74], [75]. Sexual selection has been proposed to be the major force driving this fast divergence of male reproductive traits, gene sequences, and their expression patterns [9], [10], [12]. Given that sexual reproduction is widespread among animals and sperm production appears to be present in all major phyla of metazoan animals, it raised a question whether any male-biased reproductive gene could be exempt from such selective pressure and remain conserved through extended evolutionary distances.

However, *Boule* homologs have been maintained throughout all major lineages of animals from a common eumetazoan ancestral gene and are required only for sperm development in both *Drosophila* and mice. We have shown that Boule proteins have resisted sexual selective pressure, and instead evolved under purifying selection. Though ancestral *Boule* may have also functioned in oogenesis, our findings that bilaterian *Boule* homologs tend toward male-biased expression, taken together with the similar spermatogenesis arrest phenotypes in both *Drosophila* and mouse mutants, supports the model of a common origin of bilaterian spermatogenesis.

While it remains to be seen if *Boule* homologs are restricted only to spermatogenesis or also function in the ovary, we have shown a clear case of conservation of a reproduction-specific gene across Bilateria. We found that among a broad representation of bilaterian animals, *Boule* expression was restricted to the gonads (Figure 3), indicating that it has remained reproduction-specific throughout evolution. In addition, DNA sequence analysis of multiple *Drosophila* and mammalian *Boule* homologs revealed that, unlike other reproductive proteins [11], [16], [17], *Boule* evolution has been driven not by positive selection, but by purifying selection. This establishes an unambiguous case of a reproduction-specific gene being driven predominantly by purifying selection, in two distinct animal lineages, suggesting a strong functional constraint. Interestingly, our in-depth analysis of the developmental defects in *Boule* null mice revealed a novel requirement in spermatid differentiation [61]. Such a postmeiotic function for *boule* is also likely present in *Drosophila*, though its requirement for spermatid differentiation would not have been revealed in the *boule* mutant flies due to an earlier block at meiosis [39], [61]. The previously established function of *Boule* in meiotic progression in both *Drosophila* and nematodes [39], [40] may also be conserved in mice, despite the lack of a similar meiotic defect in *Boule* null mice [61]. We proposed that *Dazl* and *Boule* may redundantly regulate meiosis, and that *Dazl* may compensate for *Boule* loss during meiosis in mice [61]. Yet despite this possibility of a partial redundancy of function with *Dazl*, mouse *Boule* has been maintained under purifying selection, further indicating that the presence of other *DAZ* family genes has had little impact on the functional constraint of *Boule*. While meiosis is fundamental to sexual reproduction and key components of meiotic machinery for chromosomal synapses and recombination are conserved from yeast to mammals [2], [76], the absence of *Boule* homologs in fungi together with the requirement of *Boule* homologs in only one sex of animals suggest that conservation of *Boule* is unlikely due to the same functional constraint that keeps components of meiotic machinery conserved. Another main functional constraint on metazoan reproduction appears to be associated with germ cell specification and maintenance. Mutations disrupting those conserved germ cell components, such as *Vasa* or *Piwi*, often result in a failure to form germ cells or a loss of germ cells before meiotic stages. Furthermore, the resulting infertility sometimes affects both males and females of the same species [21]–[23], [77]–[79]. These phenotypes differ from the sex-biased infertility of *Boule* mutations in all species examined, and the gametogenesis defects in *Boule* mutants are much less variable than those from either *Vasa* or *Piwi* mutants across species [21]–[23], [61], [78], [79]. Further characterization of the subcellular expression and molecular function of Boule will help to discern the relationship between Boule and these other highly conserved germ cell proteins.

We've shown the widespread presence of *Boule* homologs throughout bilaterian animals and the functional conservation of a reproductive-exclusive requirement among *Drosophila*, worm and mouse. This has revealed an ancient reproductive requirement in the Urbilaterian, the common ancestor of all bilaterian animals and highlights a fundamental reproductive function associated with Boule protein conserved over six hundred million years of evolution. With the identification of *Boule* and possibly more reproductive genes conserved across such large evolutionary distances, we can begin to compare the impact of sexual selection on the molecular evolution of the same components of reproductive traits in different animal lineages at both the microevolution and macroevolution levels.

## Materials and Methods

### Sequence and phylogeny analysis

For known *Boule* homologs, DNA sequences from various species were retrieved from the literature and the Genbank database. For species where the presence of *Boule* or *Dazl* homologs was unknown, we first searched the EST and cDNA database in Genbank using consensus RRM sequences of either Boule or Dazl using Tblastn and positively identified the homologs using our established criteria. The homolog sequences were further confirmed by the presence of Boule/Dazl homologs with high sequence similarity in other species within the same taxon. In the absence of EST or cDNA information, we then focused on a representative species from the same phylum whose genome had been completely sequenced. The specific genome databases were searched using the consensus Boule RRM sequences and Tblastn, and the top hits were analyzed to determine if they were *Boule* homologs based on criteria described above. The identified homologs were verified by BLASTing against the human protein database, which should identify human BOULE as the top hit sequence with highest similarity. New *Boule* and *Dazl* homologs we have identified as well as known homologs from previous publications are summarized in Table S3. Sequence alignment of RRMs and entire proteins was performed using ClustalW2 and ClustalX programs [80]. The parameters for alignment were protein Gap open penalty = 10, protein extension penalty = 0.2, and other parameters at default settings. Phylogenetic analysis was done using Mega 4.0 [81]. Ka and Ks were calculated as described [44].

### Molecular biology

RNA was extracted by Trizol from tissues and reverse transcribed for amplification of *Boule* cDNA. For tissues collected in RNA later (Applied Biosystems/Ambion, Austin TX), samples were stored at 4°C overnight, solution was removed, and the tissues were stored at −80°C for later RNA extraction. A minimum of two pairs of primers spanning the RRM region and other regions were used to confirm the expression of the *Boule* gene (Table S4). All the *Boule* amplicons were confirmed by sequencing. *Dmrt1* (*Doublesex and mab-3 related transcription factor 1*) was a testis-specific positive control in chicken [82] and *Bnd* (*Bindin*) was a testis-specific positive control in the sea urchin [83]. We used multiple sets of primers covering exons 2, 3, 4, 5 and 6, and determined that the main transcript is testis-specific (Table S4).

### Animals

Mice (*Mus musculus*) were housed and bred in a barrier facility according to the guidelines approved by the ACUC committee at Northwestern University. *Boule* mutant mice were created in the mixed background of C57B6 and 129svj. Ripe purple sea urchins (*Strongylocentrotus purpuratus*) in spawning season (May, 2009) were collected from Pacific Ocean off Carslad, California (M-REP, Carslad, California) and shipped overnight on ice to Chicago. The sex of sea urchins was determined by the presence of eggs (often a milky spill on the outside of female urchins upon arrival) and by the presence of distinct gametes in the gonad tissue biopsies. Gonads, guts and ampullae from at least three male and three female purple sea urchins were collected and either stored in Trizol for immediate RNA extraction or snap-frozen in liquid nitrogen and stored at −80°C. Chicken gonadal and other tissues were collected from euthanized White leghorn chickens at the completion of a research project approved by UIUC animal committee at the University of Illinois at Urbana-Champaign Veterinary School. Tissues from two four-year old roosters and two three-year old hens were snap-frozen in liquid nitrogen or stored directly in RNA later.

### Histology

Tissue histology was performed as described previously [35]. Testes were fixed in Bouins' solution overnight and sectioned at 5-µm thickness for hematoxylin/eosin staining. Bright field images were captured using a Leica DM 5000B compound microscope with a DFC320 camera and the Leica image capture suite software.

### Generation of a mouse *Boule* mutation

We replaced exon 3 with lacZ-neo using the NZTK2 vector (Richard Palmiter, University of Washington, Seattle, WA). We designed primers with built-in SalI sites to amplify a 2-kb left arm next to exon 3, and primers with built-in XhoI and NotI sites to amplify a 5.9-kb right arm from 129svj mouse genomic DNA. High fidelity platinum PCR kits (Invitrogen) were used to amplify the fragment with minimal PCR error. The amplified fragments were cloned into Topo vectors and later released with appropriate enzymes for subcloning into the NZTK2 vector. The clones with correct orientation of left and right arm insertions were chosen for sequencing. Sequencing of the genomic arms in the selected clones indicated that both arms had greater than 99% sequence identity with the genomic sequence. Gene targeting was performed on 200 ES cell clones (129svj E14 feeder cell-less ES cells) and four positive ES clones (1D5, 1H5, 2A4 and 2D7) were identified by the presence of both the 2-kb and 6-kb arms using primers outside each arm and on the vector. The 1D5 clone was used to inject blastocysts at the Northwestern University Transgenic Core Facility. Four chimerical mice produced lacZ-positive progeny and mice from two independent founders were used to generate mutant mice for the analysis. The phenotypes were identical among the mutant mice from two independent lines and we did not distinguish our analyses between the two lines.

## Supporting Information

Figure S1 Alignments for RNA binding domains (RRM) from insects (A) and mammals (B) reveal strong conservation among homologs. Chicken Boule RRM was added as an outgroup to mammalian domain alignment for comparison. (C) Based on the sequences from mammals and insects, the consensus RRM sequences (RNP1 and RNP 2) for Boule are likely representative of bilaterian Boule. The site of exon/intron junction is marked by the arrows and is also conserved among *Boule* homologs. The seven Drosophila species used are *D. melanogaster*, *D. sechellia*, *D. yakuba*, *D. virilis*, *D. erecta*, *D. willistoni* and *D. ananassae*. Mosquito (*Aedes aegypti*), Honeybee (*Apis mellifera*), Beetle (*Tribolium castaneum*), Wasp (*Nasonia vitripennis*), SMonkey (Squirrel Monkey, *Saimiri sciureus*), Marmoset (*Callithrix jacchus*), Tamarin (*Saquinus Oedipus*), mouse (*Mus musculus*), Rat (*Rattus norvegicus*), Lemur (*Microcebus murinus*), Human (*Homo sapiens*) Frog (*Xenopus laevis*), Chimp (*Pan troglodytes*) PChimp (Pygmy Chimp, *Pan paniscus*), RMonkey (Rhesus Monkey, *Macaca mulatta*), Hedgehog (*Erinaceus europaeus*), Microbat (*Myotis lucifugus*), GuineaPig (*Cavia porcellus*), Dog (*Canis familiaris*), Cow (*Bos Taurus*), Horse (*Equus caballus*), Bushbaby (*Otolemur garnettii*), Playtypus (*Ornithorhynchus anatinus*). Opossum (*Monodelphis domestica*), Chicken (*Gallus gallus*). Color scheme: blue– A,I,L,M,F,W,V; red—R,K; green—N,Q,S,T; pink—C; Magenta—E,D; orange—G; cyan—H,Y; yellow—P [80].
(5.46 MB TIF)

Figure S2 Dazl homologs share similar signature RRM motifs but distinct from that of Boule. (A). RRM sequence alignment of *Dazl* homologs from diverse vertebrate species. The *Dazl* RRM is two amino acids shorter than the *Boule* RRM because of a two amino acid deletion. The consensus sequence for *Dazl* RRM was generated and is distinct from *Boule* RRM (lower panel). XL Frog (*Xenopus laevis*), XT Frog (*Xenopus tropicalis*), Cow (*Bos taurus*), Human (*Homo sapiens*), PP Chimpanzee (*Pan paniscus*), PT Chimpanzee (*Pan troglodytes*), SS Monkey (squirrel monkey, *Saimiri sciureus*), RhesusMonkey (*Macaca mulatta*), Rat (*Rattus norvegicus*), mouse (*Mus musculus*), chick (*Gallus gallus*), DR Zebrafish (*Danio rerio*), OL Killifish (*Oryzias latipes*), MM Lemur (*Microcebus murinus*), SO tamarin (Saguinus Oedipus), LS Seabass (*Lates calcarifer*), RP Frog (*Rana pipiens*), CP Newt (*Cynops pyrrhogaster*), CF Dog (*Canis familiaris*), EE Hedgehog (*Erinaceus europaeus*), Opossum (*Monodelphis domestica*), Platypus (*Ornithorhynchus Anatinus*), Bushbaby (*Otolemur garnettii*), SA Shrew (*Sorex araneus*), TB Shrew (*Tupaia Belangeri*), FuguFish (*Takifugu rubripes*). (B). Alignment of Boule and Dazl RRM consensus sequences.
(5.91 MB TIF)

Figure S3 Absence in yeast and Trichoplax but presence in sea anemone of Boule homologs. (A) The yeast protein with highest similarity to the Boule consensus sequence is Hrp1, but it is not a *Boule* homolog. Sequence alignment of Boule and Hrp1 is shown. The Hrp1 RRM does not contain the characteristic amino acids of the *Boule* RRM. When the yeast Hrp1 sequence is compared to that of the fly or human genome, the fly gene with the greatest similarity is not *boule* but *hRNP*. Although yeast *Hrp1* has a sequence similarity to *Boule* in the RRM and probably represents the closest RRM protein to *Boule*, *Hrp1* is unlikely to be a *Boule* ortholog. *Boule* homologs are also absent in other single-cell eukaryotes and in plants, including *Schizosaccharomyces pombe*, *Dictyostelium discoideum* and *Arabidopsis thaliana*. We thus concluded that *Boule* homologs are restricted to animals. (B) In *Trichoplax*, the protein with highest sequence similarity to the *Boule* consensus sequence does not contain the key signature amino acids of Boule and is not a *Boule* homolog. Furthermore, the RNA binding domains of these proteins do not contain any introns, whereas *Boule* genes contain a conserved genomic structure with at least two introns separating the RRM-encoding exons at conserved junctions. Hence, a *Boule* homolog appears to be absent in the *Trichoplax* [86]. (C) There are two sea anemone proteins with high sequence similarity to the Boule consensus sequence. The sea anemone (Sa) Boule1 shows greater similarity and also shares the three internal exon-intron junctions as the consensus *Boule*, whereas sea anemone Boule2 only shares two exon-intron junctions. For phylogenetic tree construction (Figure. 1), sea anemone *Boule1* was used as the *Boule* homolog sequence. The arrows mark the sites of exon-intron junctions based on genomic sequences. RNP2 and RNP1 are underlined.
(1.84 MB TIF)

Figure S4 Real-time PCR analysis of chicken gene expression. *Dmrt1* is testis-specific control in the chicken. In the chicken, *Boule* is highly enriched in the testis, and *Dazl* is 10-fold more abundant in the testis than the ovary. *Dmrt1* RNA was not detectable in muscle, kidney or ovary, while Dazl RNA was not detectable in muscle or kidney.
(0.53 MB TIF)

Table S1 Ka/Ks ratio analysis revealed no evidence for accelerated evolution among Drosophila *boule* homologs. We performed pair-wise comparison of Ka and Ks for the entire *boule* coding sequences among seven Drosophila species (*Dmel-D. melanogaster*, *Dsec-D. sechellia*, *Dsec-D. yakuba*, *Dvir-D. virilis*, *Dere-D. erecta*, *Dwil-D. willistoni*, and *Dana-D. ananassae*). All Ka/Ks ratios were significantly below 1.
(0.05 MB DOC)

Table S2 Ka/Ks ratio analysis also failed to identify any evidence for accelerated evolution among mammalian *Boule* homologs. Molecular evolutionary analysis of the entire coding sequence in the eight representative mammalian species: Monotremes (platypus), Marsupials (opossum) and Eutherians (mouse, rat, dog, rhesus monkey, chimpanzee and human), revealed no excessive non-synonymous nucleotide changes in comparison with synonymous changes. A total of 477 nucleotides were used from each homolog. Ka/Ks ratios were all below 1, with the highest value less than 0.1. These data suggest that not only was the RRM conserved through mammalian evolution, but the entire *Boule* gene, including less conserved regions, did not undergo rapid evolution.
(0.08 MB DOC)

Table S3 Summary table listing information for all genes used in the analysis. Homologs of *Boule*, *Dazl* and *DAZ* used in this analysis are listed with species names and their sequence ID from genbank other databases.
(0.09 MB DOC)

Table S4 List of primers used for RT-PCR and qRT-PCR analyses of Chicken, Sea urchin and mouse *Boule* homologs.
(0.06 MB DOC)

Table S5 Color code table for amino acid residues in Figure 1.
(0.04 MB DOC)

Text S1 Supplemental data.
(0.04 MB DOC)

## References

[1] Ramesh MA, Malik SB, Logsdon JM (2005). A phylogenomic inventory of meiotic genes; evidence for sex in Giardia and an early eukaryotic origin of meiosis.. Curr Biol.

[2] Villeneuve AM, Hillers KJ (2001). Whence meiosis?. Cell.

[3] Charlesworth B (1991). The evolution of sex chromosome.. Science.

[4] Ellegren H, Parsch J (2007). The evolution of sex-biased genes and sex-biased gene expression.. Nat Rev Genet.

[5] Wyckoff G, Wang W, Wu C-I (2000). Rapid evolution of reproductive genes in the descent of man.. Nature.

[6] Miller GT, Pitnick S (2002). Sperm-female coevolution in Drosophila.. Science.

[7] Meiklejohn CD, Parsch J, Ranz JM, Hartl DL (2003). Rapid evolution of male-biased gene expression in Drosophila.. Proc Natl Acad Sci U S A.

[8] Marin I, Baker BS (1998). The evolutionary dynamics of sex determination.. Science.

[9] Clark NL, Aagaard JE, Swanson WJ (2006). Evolution of reproductive proteins from animals and plants.. Reproduction.

[10] Haerty W, Jagadeeshan S, Kulathinal RJ, Wong A, Ravi Ram K (2007). Evolution in the Fast Lane: Rapidly Evolving Sex-Related Genes in Drosophila.. Genetics.

[11] Nurminsky DI, Nurminskaya MV, De Aguiar D, Hartl DL (1998). Selective sweep of a newly evolved sperm-specific gene in Drosophila [see comments].. Nature.

[12] Swanson WJ, Vacquier VD (2002). The rapid evolution of reproductive proteins.. Nat Rev Genet.

[13] Zhang Y, Sturgill D, Parisi M, Kumar S, Oliver B (2007). Constraint and turnover in sex-biased gene expression in the genus Drosophila.. Nature.

[14] Good JM, Nachman MW (2005). Rates of protein evolution are positively correlated with developmental timing of expression during mouse spermatogenesis.. Mol Biol Evol.

[15] Oliver PL, Goodstadt L, Bayes JJ, Birtle Z, Roach KC (2009). Accelerated evolution of the Prdm9 speciation gene across diverse metazoan taxa.. PLoS Genet.

[16] Kalamegham R, Sturgill D, Siegfried E, Oliver B (2007). Drosophila mojoless, a retroposed GSK-3, has functionally diverged to acquire an essential role in male fertility.. Mol Biol Evol.

[17] Loppin B, Lepetit D, Dorus S, Couble P, Karr TL (2005). Origin and neofunctionalization of a Drosophila paternal effect gene essential for zygote viability.. Curr Biol.

[18] Dorus S, Busby SA, Gerike U, Shabanowitz J, Hunt DF (2006). Genomic and functional evolution of the Drosophila melanogaster sperm proteome.. Nat Genet.

[19] Chu DS, Liu H, Nix P, Wu TF, Ralston EJ (2006). Sperm chromatin proteomics identifies evolutionarily conserved fertility factors.. Nature.

[20] Zarkower D (2001). Establishing sexual dimorphism: conservation amidst diversity?. Nat Rev Genet.

[21] Aravin AA, Sachidanandam R, Girard A, Fejes-Toth K, Hannon GJ (2007). Developmentally regulated piRNA clusters implicate MILI in transposon control.. Science.

[22] Deng W, Lin H (2002). miwi, a murine homolog of piwi, encodes a cytoplasmic protein essential for spermatogenesis.. Dev Cell.

[23] Tanaka SS, Toyooka Y, Akasu R, Katoh-Fukui Y, Nakahara Y (2000). The mouse homolog of Drosophila Vasa is required for the development of male germ cells.. Genes Dev.

[24] Sada A, Suzuki A, Suzuki H, Saga Y (2009). The RNA-binding protein NANOS2 is required to maintain murine spermatogonial stem cells.. Science.

[25] Allen AK, Spradling AC (2008). The Sf1-related nuclear hormone receptor Hr39 regulates Drosophila female reproductive tract development and function.. Development.

[26] Cinalli RM, Rangan P, Lehmann R (2008). Germ cells are forever.. Cell.

[27] Extavour CGM (2007). Evolution of the bilaterian germ line: lineage origin and modulation of specification mechanisms.. Integrative & Comparative Biology.

[28] Pepling ME, de Cuevas M, Spradling AC (1999). Germline cysts: a conserved phase of germ cell development?. Trends Cell Biol.

[29] Strome S, Lehmann R (2007). Germ versus soma decisions: lessons from flies and worms.. Science.

[30] Fuller MT, Bate M, Martinez-Arias A (1993). Spermatogenesis.. Development of *Drosophila melanogaster*.

[31] Matzuk MM, Lamb DJ (2002). Genetic dissection of mammalian fertility pathways.. Nat Cell Biol.

[32] White-Cooper H, Doggett K, Ellis R, Pitnick HaB (2008). Evolution of Spermatogenesis.. Sperm Evolution: An evolutionary perspective.

[33] Kimble J, Page DC (2007). The mysteries of sexual identity. The germ cell's perspective.. Science.

[34] Roosen-Runge EC (1969). Comparative aspects of spermatogenesis.. Biol Reprod.

[35] Xu EY, Moore FL, Pera RA (2001). A gene family required for human germ cell development evolved from an ancient meiotic gene conserved in metazoans.. Proceedings of the National Academy of Sciences of the United States of America.

[36] Yen PH (2004). Putative biological functions of the DAZ family.. Int J Androl.

[37] Reijo R, Alagappan RK, Patrizio P, Page DC (1996). Severe oligozoospermia resulting from deletions of azoospermia factor gene.. Lancet.

[38] Reijo R, Lee TY, Salo P, Alagappan R, Brown LG (1995). Diverse spermatogenic defects in humans caused by Y chromosome deletions.. Nature Genetics.

[39] Eberhart CG, Maines JZ, Wasserman SA (1996). Meiotic cell cycle requirement for a fly homologue of human Deleted in Azoospermia.. Nature.

[40] Karashima T, Sugimoto A, Yamamoto M (2000). Caenorhabditis elegans homologue of the human azoospermia factor DAZ is required for oogenesis but not for spermatogenesis.. Development.

[41] Ruggiu M, Speed R, Taggart M, McKay SJ, Kilanowski F (1997). The mouse *Dazla* gene encodes a cytoplasmic protein essential for gametogenesis.. Nature.

[42] Kee K, Angeles VT, Flores M, Nguyen HN, Reijo Pera RA (2009). Human DAZL, DAZ and BOULE genes modulate primordial germ-cell and haploid gamete formation.. Nature.

[43] Yu Z, Ji P, Cao J, Zhu S, Li Y (2009). Dazl promotes germ cell differentiation from embryonic stem cells.. J Mol Cell Biol.

[44] Xu EY, Lee DF, Klebes A, Turek PJ, Kornberg TB (2003). Human BOULE gene rescues meiotic defects in infertile flies.. Human Molecular Genetics.

[45] Otori M, Karashima T, Yamamoto M (2006). The Caenorhabditis elegans homologue of deleted in azoospermia is involved in the sperm/oocyte switch.. Mol Biol Cell.

[46] Houston DW, Zhang J, Maines JZ, Wasserman SA, King ML (1998). A Xenopus DAZ-like gene encodes an RNA component of germ plasm and is a functional homologue of Drosophila boule.. Development.

[47] Houston DW, King ML (2000). A critical role for *Xdazl*, a germ plasm-localized RNA, in the differentiation of primordial germ cells in *Xenopus*.. Development.

[48] Bandziulis RJ, Swanson MS, Dreyfuss G (1989). RNA-binding proteins as developmental regulators.. Genes Dev.

[49] Putnam NH, Srivastava M, Hellsten U, Dirks B, Chapman J (2007). Sea Anemone Genome Reveals Ancestral Eumetazoan Gene Repertoire and Genomic Organization.. Science.

[50] Bachvarova RF, Crother BI, Manova K, Chatfield J, Shoemaker CM (2009). Expression of Dazl and Vasa in turtle embryos and ovaries: evidence for inductive specification of germ cells.. Evol Dev.

[51] van de Lavoir MC, Diamond JH, Leighton PA, Mather-Love C, Heyer BS (2006). Germline transmission of genetically modified primordial germ cells.. Nature.

[52] Maegawa S, Yasuda K, Inoue K (1999). Maternal mRNA localization of zebrafish *DAZ-like* gene.. Mech Dev.

[53] Xu H, Li Z, Li M, Wang L, Hong Y (2009). Boule is present in fish and bisexually expressed in adult and embryonic germ cells of medaka.. PLoS One.

[54] Venkatesh B, Kirkness EF, Loh YH, Halpern AL, Lee AP (2007). Survey sequencing and comparative analysis of the elephant shark (Callorhinchus milii) genome.. PLoS Biol.

[55] Saxena R, Brown LG, Hawkins T, Alagappan RK, Skaletsky H (1996). The DAZ gene cluster on the human Y chromosome arose from an autosomal gene that was transposed, repeatedly amplified and pruned.. Nature Genetics.

[56] Clark AG, Eisen MB, Smith DR, Bergman CM, Oliver B (2007). Evolution of genes and genomes on the Drosophila phylogeny.. Nature.

[57] Juliano CE, Voronina E, Stack C, Aldrich M, Cameron AR (2006). Germ line determinants are not localized early in sea urchin development, but do accumulate in the small micromere lineage.. Dev Biol.

[58] Dorfman DM, Genest DR, Reijo Pera RA (1999). Human DAZL1 encodes a candidate fertility factor in women that localizes to the prenatal and postnatal germ cells.. Human Reproduction.

[59] Hoopfer ED, Penton A, Watts RJ, Luo L (2008). Genomic analysis of Drosophila neuronal remodeling: a role for the RNA-binding protein Boule as a negative regulator of axon pruning.. J Neurosci.

[60] Zhang Q, Li J, Li Q, Li X, Liu Z (2009). Cloning and characterization of the gene encoding the bovine BOULE protein.. Mol Genet Genomics.

[61] Vangompel MJ, Xu EY (2010). A novel requirement in mammalian spermatid differentiation for the DAZ-family protein Boule.. Hum Mol Genet.

[62] Brill JA, Hime GR, Scharer-Schuksz M, Fuller MT (2000). A phospholipid kinase regulates actin organization and intercellular bridge formation during germline cytokinesis.. Development.

[63] Fawcett DW, Ito S, Slautterback D (1959). The occurrence of intercellular bridges in groups of cells exhibiting synchronous differentiation.. J Biophys Biochem Cytol.

[64] Greenbaum MP, Ma L, Matzuk MM (2007). Conversion of midbodies into germ cell intercellular bridges.. Dev Biol.

[65] Hime GR, Brill JA, Fuller MT (1996). Assembly of ring canals in the male germ line from structural components of the contractile ring.. Journal of Cell Science.

[66] Hedges SB (2002). The origin and evolution of model organisms.. Nat Rev Genet.

[67] Knoll AH, Carroll SB (1999). Early Animal Evolution: Emerging Views from Comparative Biology and Geology.. Science.

[68] Joiner M-LA, Wu C-F (2004). Nervous system function for the testis RNA-binding protein Boule in Drosophila.. Journal of Neurogenetics.

[69] Fuller MT (1998). Genetic control of cell proliferation and differentiation in Drosophila spermatogenesis.. Seminars in Cell and Developmental Biology.

[70] Levine MT, Jones CD, Kern AD, Lindfors HA, Begun DJ (2006). Novel genes derived from noncoding DNA in Drosophila melanogaster are frequently X-linked and exhibit testis-biased expression.. Proc Natl Acad Sci U S A.

[71] Vibranovski MD, Lopes HF, Karr TL, Long M (2009). Stage-specific expression profiling of Drosophila spermatogenesis suggests that meiotic sex chromosome inactivation drives genomic relocation of testis-expressed genes.. PLoS Genet.

[72] Braun RE (2001). Packaging paternal chromosomes with protamine.. Nat Genet.

[73] Eirin-Lopez JM, Ausio J (2009). Origin and evolution of chromosomal sperm proteins.. Bioessays.

[74] Cho C, Willis WD, Goulding EH, Jung-Ha H, Choi YC (2001). Haploinsufficiency of protamine-1 or -2 causes infertility in mice.. Nat Genet.

[75] Rathke C, Baarends WM, Jayaramaiah-Raja S, Bartkuhn M, Renkawitz R (2007). Transition from a nucleosome-based to a protamine-based chromatin configuration during spermiogenesis in Drosophila.. J Cell Sci.

[76] Keeney S (2001). Mechanism and control of meiotic recombination initiation.. Curr Top Dev Biol.

[77] Hay B, Jan LY, Jan YN (1990). Localization of vasa, a component of Drosophila polar granules, in maternal-effect mutants that alter embryonic anteroposterior polarity.. Development.

[78] Lasko PaMA (1990). Posterior localization of vsa protein correlates with, but is not sufficient for, pole cell development.. Genes and Development.

[79] Lin H, Spradling AC (1997). A novel goup of pumilio mutations affects the asymmetric division of germline stem cells in the Drosophila ovary.. Development.

[80] Larkin MA, Blackshields G, Brown NP, Chenna R, McGettigan PA (2007). Clustal W and Clustal X version 2.0.. Bioinformatics.

[81] Tamura K, Dudley J, Nei M, Kumar S (2007). MEGA4: Molecular Evolutionary Genetics Analysis (MEGA) software version 4.0.. Mol Biol Evol.

[82] Shan Z, Nanda I, Wang Y, Schmid M, Vortkamp A (2000). Sex-specific expression of an evolutionarily conserved male regulatory gene, DMRT1, in birds.. Cytogenet Cell Genet.

[83] Cameron RA, Minor JE, Nishioka D, Britten RJ, Davidson EH (1990). Locale and level of bindin mRNA in maturing testis of the sea urchin, Strongylocentrotus purpuratus.. Dev Biol.

[84] Roosen-Rung EC (1977). The process of spermatogenesis in animals.

[85] Futuyma DJ (1986). Evolutionary Biology.

[86] Srivastava M, Begovic E, Chapman J, Putnam NH, Hellsten U (2008). The Trichoplax genome and the nature of placozoans.. Nature.

