# Supplementary figures and images for: Widespread Presence of Human BOULE Homologs among Animals and Conservation of Their Ancient Reproductive Function
Source: PLoS Genet. 2010 Jul 15;6(7):e1001022. doi: 10.1371/journal.pgen.1001022 (PMC2904765; doi:10.1371/journal.pgen.1001022)

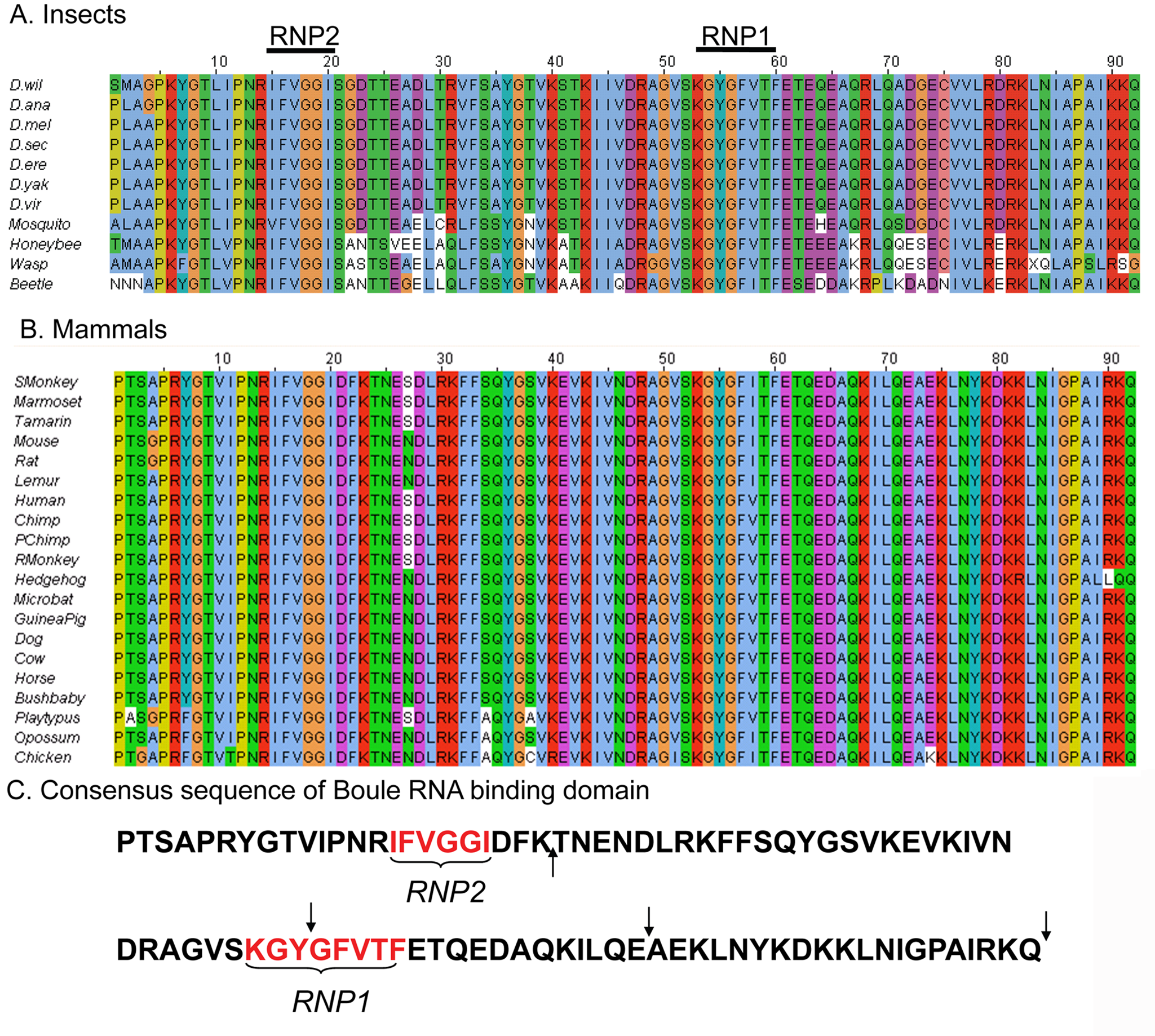

Supplement: Figure S1 — Alignments for RNA binding domains (RRM) from insects (A) and mammals (B) reveal strong conservation among homologs. Chicken Boule RRM was added as an outgroup to mammalian domain alignment for comparison. (C) Based on the sequences from mammals and insects, the consensus RRM sequences (RNP1 and RNP 2) for Boule are likely representative of bilaterian Boule. The site of exon/intron junction is marked by the arrows and is also conserved among Boule homologs. The seven Drosophila species used are D. melanogaster, D. sechellia, D. yakuba, D. virilis, D. erecta, D. willistoni and D. ananassae. Mosquito (Aedes aegypti), Honeybee (Apis mellifera), Beetle (Tribolium castaneum), Wasp (Nasonia vitripennis), SMonkey (Squirrel Monkey, Saimiri sciureus), Marmoset (Callithrix jacchus), Tamarin (Saquinus Oedipus), mouse (Mus musculus), Rat (Rattus norvegicus), Lemur (Microcebus murinus), Human (Homo sapiens) Frog (Xenopus laevis), Chimp (Pan troglodytes) PChimp (Pygmy Chimp, Pan paniscus), RMonkey (Rhesus Monkey, Macaca mulatta), Hedgehog (Erinaceus europaeus), Microbat (Myotis lucifugus), GuineaPig (Cavia porcellus), Dog (Canis familiaris), Cow (Bos Taurus), Horse (Equus caballus), Bushbaby (Otolemur garnettii), Playtypus (Ornithorhynchus anatinus). Opossum (Monodelphis domestica), Chicken (Gallus gallus). Color scheme: blue– A,I,L,M,F,W,V; red—R,K; green—N,Q,S,T; pink—C; Magenta—E,D; orange—G; cyan—H,Y; yellow—P [80]. (5.46 MB TIF) [file pgen.1001022.s001.tif]

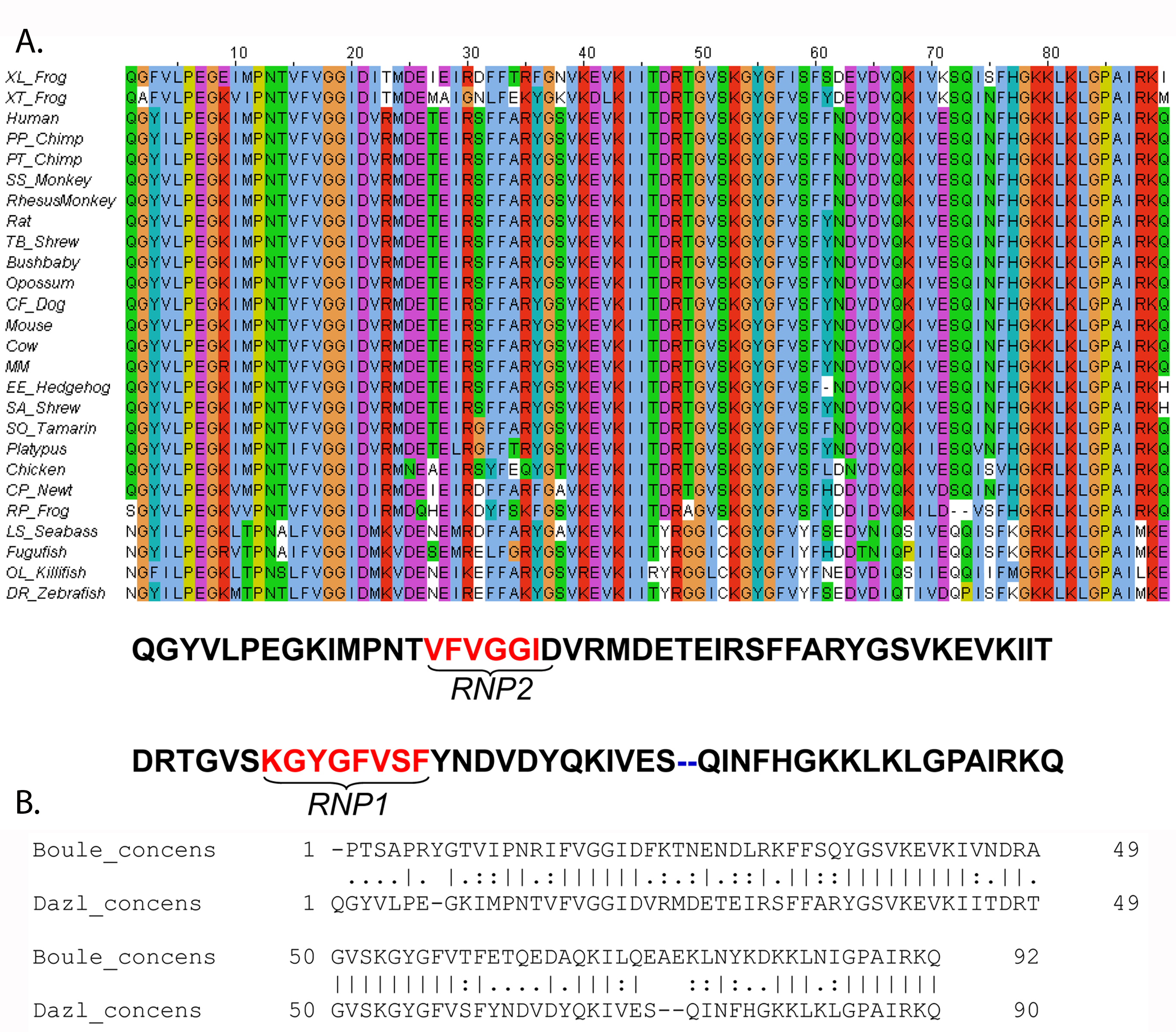

Supplement: Figure S2 — Dazl homologs share similar signature RRM motifs but distinct from that of Boule. (A). RRM sequence alignment of Dazl homologs from diverse vertebrate species. The Dazl RRM is two amino acids shorter than the Boule RRM because of a two amino acid deletion. The consensus sequence for Dazl RRM was generated and is distinct from Boule RRM (lower panel). XL Frog (Xenopus laevis), XT Frog (Xenopus tropicalis), Cow (Bos taurus), Human (Homo sapiens), PP Chimpanzee (Pan paniscus), PT Chimpanzee (Pan troglodytes), SS Monkey (squirrel monkey, Saimiri sciureus), RhesusMonkey (Macaca mulatta), Rat (Rattus norvegicus), mouse (Mus musculus), chick (Gallus gallus), DR Zebrafish (Danio rerio), OL Killifish (Oryzias latipes), MM Lemur (Microcebus murinus), SO tamarin (Saguinus Oedipus), LS Seabass (Lates calcarifer), RP Frog (Rana pipiens), CP Newt (Cynops pyrrhogaster), CF Dog (Canis familiaris), EE Hedgehog (Erinaceus europaeus), Opossum (Monodelphis domestica), Platypus (Ornithorhynchus Anatinus), Bushbaby (Otolemur garnettii), SA Shrew (Sorex araneus), TB Shrew (Tupaia Belangeri), FuguFish (Takifugu rubripes). (B). Alignment of Boule and Dazl RRM consensus sequences. (5.91 MB TIF) [file pgen.1001022.s002.tif]

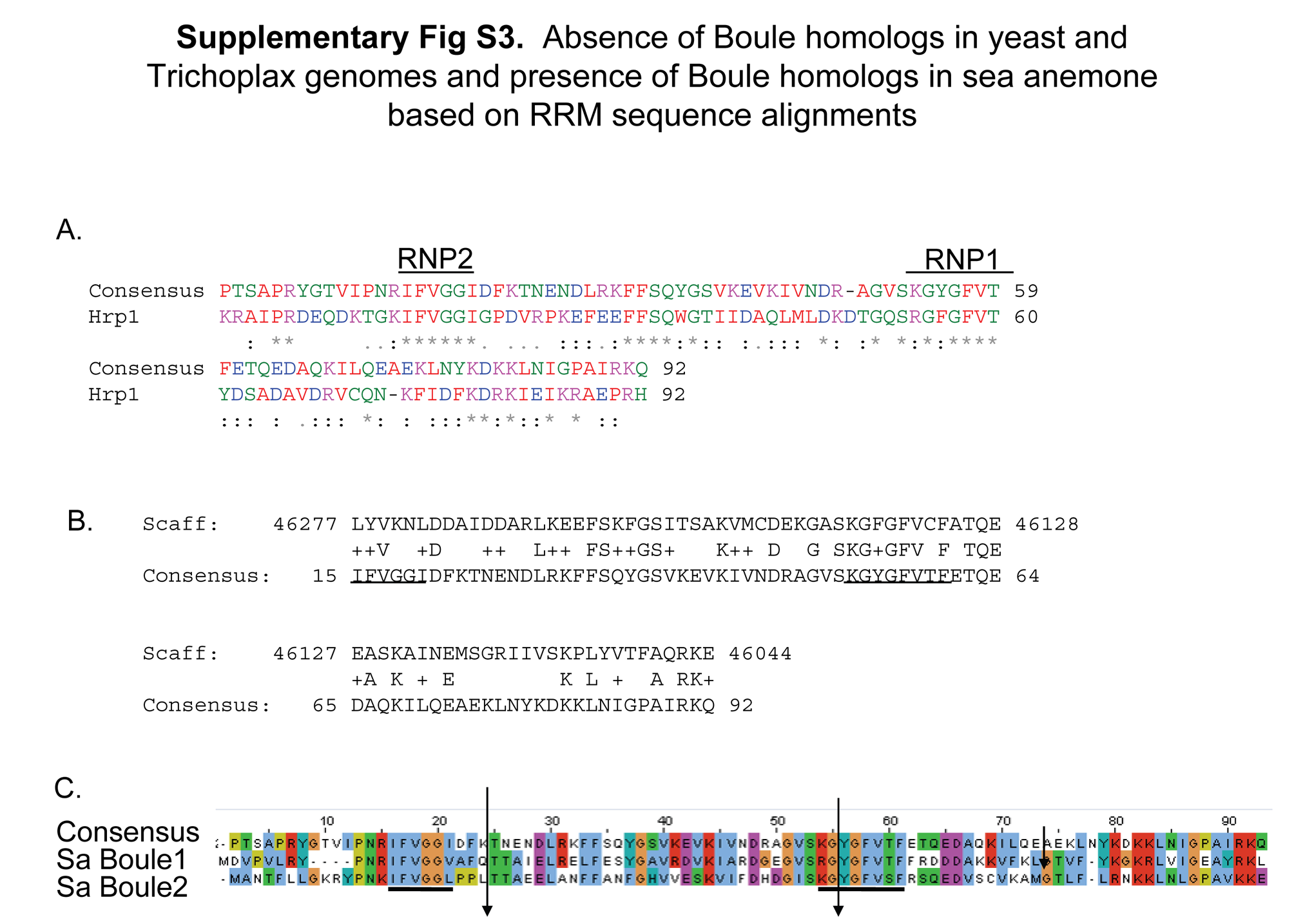

Supplement: Figure S3 — Absence in yeast and Trichoplax but presence in sea anemone of Boule homologs. (A) The yeast protein with highest similarity to the Boule consensus sequence is Hrp1, but it is not a Boule homolog. Sequence alignment of Boule and Hrp1 is shown. The Hrp1 RRM does not contain the characteristic amino acids of the Boule RRM. When the yeast Hrp1 sequence is compared to that of the fly or human genome, the fly gene with the greatest similarity is not boule but hRNP. Although yeast Hrp1 has a sequence similarity to Boule in the RRM and probably represents the closest RRM protein to Boule, Hrp1 is unlikely to be a Boule ortholog. Boule homologs are also absent in other single-cell eukaryotes and in plants, including Schizosaccharomyces pombe, Dictyostelium discoideum and Arabidopsis thaliana. We thus concluded that Boule homologs are restricted to animals. (B) In Trichoplax, the protein with highest sequence similarity to the Boule consensus sequence does not contain the key signature amino acids of Boule and is not a Boule homolog. Furthermore, the RNA binding domains of these proteins do not contain any introns, whereas Boule genes contain a conserved genomic structure with at least two introns separating the RRM-encoding exons at conserved junctions. Hence, a Boule homolog appears to be absent in the Trichoplax [86]. (C) There are two sea anemone proteins with high sequence similarity to the Boule consensus sequence. The sea anemone (Sa) Boule1 shows greater similarity and also shares the three internal exon-intron junctions as the consensus Boule, whereas sea anemone Boule2 only shares two exon-intron junctions. For phylogenetic tree construction (Figure. 1), sea anemone Boule1 was used as the Boule homolog sequence. The arrows mark the sites of exon-intron junctions based on genomic sequences. RNP2 and RNP1 are underlined. (1.84 MB TIF) [file pgen.1001022.s003.tif]

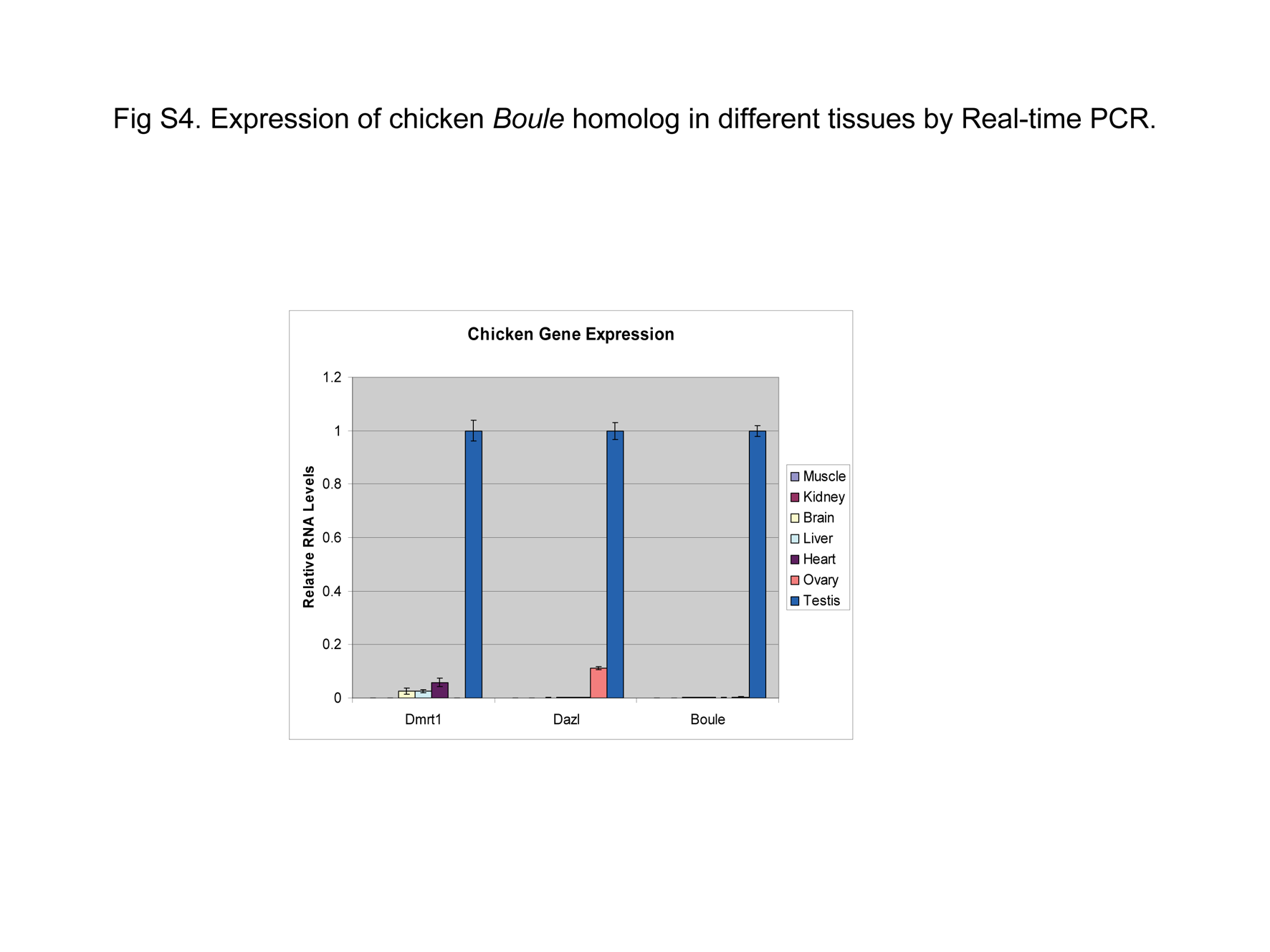

Supplement: Figure S4 — Real-time PCR analysis of chicken gene expression. Dmrt1 is testis-specific control in the chicken. In the chicken, Boule is highly enriched in the testis, and Dazl is 10-fold more abundant in the testis than the ovary. Dmrt1 RNA was not detectable in muscle, kidney or ovary, while Dazl RNA was not detectable in muscle or kidney. (0.53 MB TIF) [file pgen.1001022.s004.tif]
