# Supplementary material for: Widespread Presence of Human BOULE Homologs among Animals and Conservation of Their Ancient Reproductive Function
Source: PLoS Genet. 2010 Jul 15;6(7):e1001022. doi: 10.1371/journal.pgen.1001022 (PMC2904765; doi:10.1371/journal.pgen.1001022)
Supplement: Table S1 — Ka/Ks ratio analysis revealed no evidence for accelerated evolution among Drosophila boule homologs. We performed pair-wise comparison of Ka and Ks for the entire boule coding sequences among seven Drosophila species (Dmel-D. melanogaster, Dsec-D. sechellia, Dsec-D. yakuba, Dvir-D. virilis, Dere-D. erecta, Dwil-D. willistoni, and Dana-D. ananassae). All Ka/Ks ratios were significantly below 1. (0.05 MB DOC) [file pgen.1001022.s005.doc]

**Table S1. Molecular evolutionary analyses of *boule* homologs in *Drosophila* species.**

| **Species** | **Ka** | **Ks** | **Ka:Ks** |
| --- | --- | --- | --- |
| Dmel/Dsec | 0.003 | 0.066 | 0.045 |
| Dmel/Dere | 0.005 | 0.156 | 0.032 |
| Dmel/Dyak | 0.003 | 0.154 | 0.016 |
| Dmel/Dvir | 0.036 | 0.600 | 0.060 |
| Dmel/Dwil | 0.057 | 0.998 | 0.058 |
| Dmel/Dana | 0.018 | 0.525 | 0.035 |
| Dsec/Dere | 0.005 | 0.148 | 0.037 |
| Dsec/Dyak | 0.003 | 0.133 | 0.022 |
| Dsec/Dvir | 0.036 | 0.656 | 0.055 |
| Dsec/Dwil | 0.058 | 0.841 | 0.069 |
| Dsec/Dana | 0.019 | 0.493 | 0.038 |
| Dere/Dyak | 0.002 | 0.107 | 0.023 |
| Dere/Dvir | 0.039 | 0.571 | 0.068 |
| Dere/Dwil | 0.057 | 0.816 | 0.070 |
| Dere/Dana | 0.021 | 0.428 | 0.050 |
| Dyak/Dvir | 0.033 | 0.641 | 0.052 |
| Dyak/Dwil | 0.055 | 0.953 | 0.057 |
| Dyak/Dana | 0.016 | 0.476 | 0.033 |
| Dvir/Dwil | 0.039 | 0.804 | 0.048 |
| Dvir/Dana | 0.022 | 0.679 | 0.032 |
| Dwil/Dana | 0.041 | 0.708 | 0.058 |

A total of 510 nucleotides were compared.
