# Supplementary material for: Widespread Presence of Human BOULE Homologs among Animals and Conservation of Their Ancient Reproductive Function
Source: PLoS Genet. 2010 Jul 15;6(7):e1001022. doi: 10.1371/journal.pgen.1001022 (PMC2904765; doi:10.1371/journal.pgen.1001022)
Supplement: Table S2 — Ka/Ks ratio analysis also failed to identify any evidence for accelerated evolution among mammalian Boule homologs. Molecular evolutionary analysis of the entire coding sequence in the eight representative mammalian species: Monotremes (platypus), Marsupials (opossum) and Eutherians (mouse, rat, dog, rhesus monkey, chimpanzee and human), revealed no excessive non-synonymous nucleotide changes in comparison with synonymous changes. A total of 477 nucleotides were used from each homolog. Ka/Ks ratios were all below 1, with the highest value less than 0.1. These data suggest that not only was the RRM conserved through mammalian evolution, but the entire Boule gene, including less conserved regions, did not undergo rapid evolution. (0.08 MB DOC) [file pgen.1001022.s006.doc]

**Table S2. Molecular evolutionary analyses of mammalian *Boule*.**

| **Species** | **Ka** | **Ks** | **Ka:Ks** |
| --- | --- | --- | --- |
| Orangutan/Chimp | 0 | 0.02797 | 0 |
| Orangutan/Macaque | 0 | 0.03045 | 0 |
| Orangutan/Human | 0 | 0.0347 | 0 |
| Orangutan/Mouse | 0.01472 | 0.21787 | 0.068 |
| Orangutan/Mouse | 0.0089 | 0.0893 | 0.1 |
| Orangutan/Rat | 0.01204 | 0.23522 | 0.051 |
| Orangutan/Dog | 0.00312 | 0.08772 | 0.036 |
| Orangutan/Opossum | 0.01471 | 0.224 | 0.066 |
| Orangutan/Platypus | 0.04734 | 0.82555 | 0.057 |
| Chimp/Macaque | 0 | 0.03719 | 0 |
| Chimp/Human | 0 | 0.00653 | 0 |
| Chimp/Mouse | 0.01472 | 0.22727 | 0.065 |
| Chimp/Mouse | 0.0089 | 0.09784 | 0.091 |
| Chimp/Rat | 0.01204 | 0.24465 | 0.049 |
| Chimp/Dog | 0.00312 | 0.09565 | 0.033 |
| Chimp/Opossum | 0.01471 | 0.23375 | 0.063 |
| Chimp/Platypus | 0.04734 | 0.81125 | 0.058 |
| Macaque/Human | 0 | 0.04413 | 0 |
| Macaque/Mouse | 0.01472 | 0.2145 | 0.069 |
| Macaque/Mouse | 0.0089 | 0.08698 | 0.102 |
| Macaque/Rat | 0.01204 | 0.21303 | 0.057 |
| Macaque/Dog | 0.00312 | 0.08459 | 0.037 |
| Macaque/Opossum | 0.01471 | 0.20175 | 0.073 |
| Macaque/Platypus | 0.04734 | 0.86702 | 0.055 |
| Human/Mouse | 0.01472 | 0.21787 | 0.068 |
| Human/Mouse | 0.0089 | 0.10672 | 0.083 |
| Human/Rat | 0.01204 | 0.25449 | 0.047 |
| Human/Dog | 0.00312 | 0.10389 | 0.03 |
| Human/Opossum | 0.01471 | 0.24395 | 0.06 |
| Human/Platypus | 0.04734 | 0.84195 | 0.056 |
| Mouse/Mouse | 0.00575 | 0.23734 | 0.024 |
| Mouse/Rat | 0.00262 | 0.15461 | 0.017 |
| Mouse/Dog | 0.01445 | 0.22502 | 0.064 |
| Mouse/Opossum | 0.01683 | 0.39828 | 0.042 |
| Mouse/Platypus | 0.05639 | 1.03806 | 0.054 |
| Mouse/Rat | 0.00839 | 0.25372 | 0.033 |
| Mouse/Dog | 0.00575 | 0.08536 | 0.067 |
| Mouse/Opossum | 0.01102 | 0.24019 | 0.046 |
| Mouse/Platypus | 0.05142 | 0.85633 | 0.06 |
| Rat/Dog | 0.01178 | 0.22166 | 0.053 |
| Rat/Opossum | 0.01417 | 0.36629 | 0.039 |
| Rat/Platypus | 0.05909 | 0.94895 | 0.062 |
| Dog/Opossum | 0.01153 | 0.23755 | 0.049 |
| Dog/Platypus | 0.05069 | 0.77438 | 0.065 |
| Opossum/Platypus | 0.05323 | 0.66195 | 0.08 |
