# Supplementary material for: Widespread Presence of Human BOULE Homologs among Animals and Conservation of Their Ancient Reproductive Function
Source: PLoS Genet. 2010 Jul 15;6(7):e1001022. doi: 10.1371/journal.pgen.1001022 (PMC2904765; doi:10.1371/journal.pgen.1001022)
Supplement: Table S3 — Summary table listing information for all genes used in the analysis. Homologs of Boule, Dazl and DAZ used in this analysis are listed with species names and their sequence ID from genbank other databases. (0.09 MB DOC) [file pgen.1001022.s007.doc]

Table S3 List of *Boule, Dazl* and *DAZ* Homologs used in the phylogenetic analysis.

| **Homologs** | **Species/Common Name** | **Scientific Name of Species** | **genbank ID/DNA** |
| --- | --- | --- | --- |
| Boule | Acorn worm | *Saccoglossus kowalevskii* | ACQM01051524 contig |
| Boule | Beetle | *Tribolium castaneum* | XP_972674 |
| Boule | Chimp | *Pan troglodytes* | ENSPTRT00000023673 |
| Boule | Chicken | *Gallus Gallus* | XM_421917 |
| Boule | Dog | *Canis familiaris* | ENSCAFT00000017417 |
| Boule | Elephant Shark | *Callorhinchus milli* | [AAVX01290402.1](http://blast.fugu-sg.org/cgi-bin/scaff_shark.pl?scaffold=AAVX01290402.1) |
| Boule | Flatworm | *Schistosoma japonicum* | BU791962 |
| Boule | Flatworm | *Schistosoma mansoni* | XM_002575473 |
| Boule | Fruitfly | *D. melanogaster* | BE976181 |
| Boule | Fruitfly | *Drosophila virilis* | XM_002046229 |
| Boule | Green crab | *Carcinus Maenas* | DW249210 |
| Boule | Honeybee | *Apis mellifera* | XM_001122634 |
| Boule | Human | *Homo sapiens* | OTTHUMT00000256107 |
| Boule | Lancelet | *Branchiostoma floridae* | BW807462 |
| Boule | Lamprey | *Petromyzon marinus* | Contigs 9667/60909 |
| Boule | Leech | *Helobdella robusta* | EY313120 |
| Boule | Lobster | *Homarus americanus* | CN853821 |
| Boule | Macaque | *Macaca mulatta* | ENSMMUT00000033507 |
| Boule | mosquito | *Anopheles gambiae* | XM_315505 |
| Boule | Mouse | *Mus musculus* | OTTMUST00000074608 |
| Boule | Nematode | *C. briggsae* | CBG02404 |
| Boule | Nematode | *C. elegans* | F56D1.7 |
| Boule | Opossum | *Monodelphis domestica* | ENSMODT00000015724 |
| Boule | Orangutan | *Pongo pygmaeus* | ENSPPYT00000015171 |
| Boule | Playtypus | *Ornithorhynchus anatinus* | ENSOANT00000029092 |
| Boule | Rat | *Rattus norvegicus* | ENSRNOT00000021299 |
| Boule | Rainbow trout | *Oncorhynchus mykiss* | BX877538.3 |
| Boule | Red Flour Beetle | *Tribolium castaneum* | XM_969351.2 |
| Boule | Sea anemone | *Nematostella vectensis* | XM_001637198.1 |
| Boule | Sea Anemone | *Nematostella vectensis* | XM_001635170 |
| Boule | Sea Bass | *Dicentrarchus labrax* | FM006586.1 |
| Boule | Snail | *Biomphalaria glabrata* | EV821571.2 |
| Boule | Sea squirt | *Ciona intestinalis* | [XM_002124582.1](http://www.ncbi.nlm.nih.gov/nuccore/198423972) |
| Boule | Sea urchin | *Strongylocentrotus purpuratus* | XM_781752.1 |
| Boule | Tamarin | *Saquinus oedipus* | CAG30557 |
| Boule | Wasp | *Nasonia vitripennis* | XM_001599348 |
|  |  |  |  |
| DAZ | Human | *Homo Sapiens* | NM_020420.2 |
| Daz | Macaque | *Macaca Mulatta* | FJ648738 |
|  |  |  |  |
| Dazl | Platypus | *Ornithorhynchus anatinus* | XM_001514679 |
| Dazl | Chicken | *Gallus gallus* | NM_204218 |
| Dazl | Chimp | *Pan troglodytes* | XM_516314.2 |
| Dazl | Frog | *Xenopus laevis* | NM_001088303 |
| Dazl | Human | *Homo_sapiens* | Hs.131179 |
| Dazl | Lemur | *Canis familiaris* | XM_534251 |
| Dazl | Macaque | *Macaca mulatta* | XM_001084406 |
| Dazl | Mouse | *Mus musculus* | NM_010021 |
| Dazl | Puffer fish | *Takifugu rubripes* | SINFRUT00000159047 |
| Dazl | Zebra fish | *Danio rerio* | NM_131524 |
|  |  |  |  |
| hrp1 | Yeast | *Saccharomyces cerevisiae* | NC_001147 |
|  |  |  |  |
