# Supplementary material for: Widespread Presence of Human BOULE Homologs among Animals and Conservation of Their Ancient Reproductive Function
Source: PLoS Genet. 2010 Jul 15;6(7):e1001022. doi: 10.1371/journal.pgen.1001022 (PMC2904765; doi:10.1371/journal.pgen.1001022)
Supplement: Table S4 — List of primers used for RT-PCR and qRT-PCR analyses of Chicken, Sea urchin and mouse Boule homologs. (0.06 MB DOC) [file pgen.1001022.s008.doc]

**Table S4** Primers used in the RT-PCR and qRT-PCR analyses.

Primers for RT-PCR (gels)

| Gene | Forward | Reverse | Product Size |
| --- | --- | --- | --- |
| CHICKEN |  |  |  |
| Gg Actin | CACAGATCATGTTTGAGACCTT | CATCACAATACCAGTGGTACG | 101 bp |
| Gg Dazl | GTCAACAACCTGCCAAGGAT | TCCACATTGTCCAGGAATGA | 207 bp |
| Gg Dmrt1 | CGCGTCTGCCCAAGTG | GATGGAAGGGATGTCCTGAATGA | 367 bp |
| Gg Bol | CACGCTCTGTTTCCAGTTCA | GGGTGCTTGAACTCCATGAT | 278 bp |
| URCHIN |  |  |  |
| Sp Ubq | CACAGGCAAGACCATCACAC | GAGAGAGTGCGACCATCCTC | 110 bp |
| Sp Bnd | TCAGAGGATGGCGTTAGCTT | CTGGAGGTAACGCACCAAAT | 616 bp |
| Sp Bol | ACGCCAGAGGAAGCACATAG | GGTAGGCAAACTGTTGATGTTG | 332 bp |
| MOUSE |  |  |  |
| 2mINSF-E9R | agagctggagtgtccaaagg | cagtggaggaggaacacagc | 501bp |
| 2mINSF-Ex12R | agagctggagtgtccaaagg | gtgtgggcttcgagaatagc | 712 bp and 847bp |

Additional Primers for qRT-PCR

| Gene | Forward | Reverse | Product Size |
| --- | --- | --- | --- |
| Gg Dazl | CGTGCAGCCTCAAGCTGTGGT | TGTGTGGGCTCTGGGACAGCA | 255 bp |
| Sp Actin | CAAGGTGTCATGGTCGGCAT | GGGTACTTCAGGGTGAGGATAC | 75 bp |

Other mouse *Boule* exonic primers used to characterize *Boule* transcripts.

Forward primers:

| **primer** | **sequence (5’-3’)** | **location** |
| --- | --- | --- |
| mBolB-Ex1F | acgatgacccgagagaacc | exon 1 |
| mBol-Ex2F | aacaagtggcccaagatacg | exon 2 |
| 2mINSF | agagctggagtgtccaaagg | exon 3 |
| mBol-Ex5F | aactcaacattggtccagca | exon 5 |
| mBol-699F | aacttctgtcccaccatcttg | exon 6 |
| mBol-744F | tcctgtgatggtggctcag | exon 7 |
| mBol-Ex9F | cctgcttcttctgctccatt | exon 9 |
| mBol-Ex10F | ttgctatgcctgcacctatg | exon 10 |

Reverse primers:

| **primer** | **sequence (5’-3’)** | **location** |
| --- | --- | --- |
| mBol-Ex4R | tttgtgcatcttcttgagtttca | exon 4 |
| mBol-945R | cagtggaggaggaacacagc | exon 9 |
| mBol-Ex11R_est | cacacaaggatcaatggacaa | exon 11 |
| mBolE-Ex12R | gtgtgggcttcgagaatagc | exon 12 |
| lacZ1’ | cgacgttgtaaaacgacgggatc | lacZ on KO vector |
