# Supplementary material for: Widespread Presence of Human BOULE Homologs among Animals and Conservation of Their Ancient Reproductive Function
Source: PLoS Genet. 2010 Jul 15;6(7):e1001022. doi: 10.1371/journal.pgen.1001022 (PMC2904765; doi:10.1371/journal.pgen.1001022)
Supplement: Table S5 — Color code table for amino acid residues in Figure 1. (0.04 MB DOC) [file pgen.1001022.s009.doc]

**Supplementary Table S5**

Color code for Figure 1

| Color | residues |
| --- | --- |
| Yellow | A, F, I, L, M, V |
| Red | K, R |
| Green | N, Q, S, T, W |
| Aqua | D, E |
| Fuchsia | G |
| Teal | H |
| Olive | C |
| Lime | Y |
| Blue | P |
