# Supplementary material for: Widespread Presence of Human BOULE Homologs among Animals and Conservation of Their Ancient Reproductive Function
Source: PLoS Genet. 2010 Jul 15;6(7):e1001022. doi: 10.1371/journal.pgen.1001022 (PMC2904765; doi:10.1371/journal.pgen.1001022)
Supplement: Text S1 — Supplemental data. (0.04 MB DOC) [file pgen.1001022.s010.doc]

Supplementary Materials

Supplementary Data

**Genomic structure of mammalian *Boule.*** To characterize mouse *Boule*, we analyzed multiple *Boule* cDNA clones and RT-PCR products from mouse gonads and revealed that mouse *Boule* contains a total of 12 exons located within 115 kb of chromosome 1. While exon 1 to exon 11 were well conserved compared with the human *BOULE* genomic structure, exon 12 had not been identified in any human *BOULE* cDNA [1]. We identified a conserved region corresponding to mouse exon 12 downstream of human exon 11 (83% sequence identity), suggesting that exon 12 may be also part of the human *BOULE* gene. As in human *BOULE*, multiple alternatively spliced isoforms exist in the mouse. In addition to 5’ end alternative splicing of exon 1 (exon 1A and exon 1B), alternative splicing also occurs at the 3’ end of the gene to skip exon 11 [1] (Fig. 3C). This alternative splicing pattern resembles that of the *boule* gene in *Drosophila*, which contains a total of 10 exons with alternative splicing at the 5’ and 3’ ends [2]. In contrast, worm *daz-1* consists of six exons with only the exon 2 and exon 3 junctions shared with the consensus genomic structure. The overall genomic structure of worm *daz-1*, like the sequence of its RRM, is more divergent from the consensus features of eumetazoan *Boule* than either fly *boule* or mouse *Boule* (Fig. 1A).

Supplementary Information References

1. Kostova E, Yeung CH, Luetjens CM, Brune M, Nieschlag E, et al. (2007) Association of three isoforms of the meiotic BOULE gene with spermatogenic failure in infertile men. Mol Hum Reprod 13: 85-93.

2. Hoopfer ED, Penton A, Watts RJ, Luo L (2008) Genomic analysis of Drosophila neuronal remodeling: a role for the RNA-binding protein Boule as a negative regulator of axon pruning. J Neurosci 28: 6092-6103.
